# Steering the Catalytic Properties of Intermetallic Compounds and Alloys in Reforming Reactions by Controlled *in Situ* Decomposition and Self-Activation

**DOI:** 10.1021/acscatal.1c00718

**Published:** 2021-04-16

**Authors:** Simon Penner, Parastoo Delir Kheyrollahi Nezhad

**Affiliations:** † Department of Physical Chemistry, University of Innsbruck, Innrain 52c, A-6020 Innsbruck, Austria; ‡ Reactor and Catalyst Research Lab, Department of Chemical Engineering, University of Tabriz, Tabriz, Iran

**Keywords:** methanol steam reforming, methane dry reforming, dynamics, thermodynamic stability, phase boundary, carbon reactivity, water activation

## Abstract

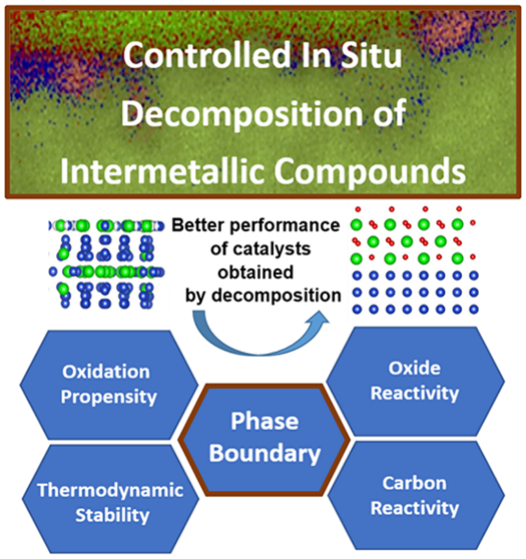

Based on the increasing importance of intermetallic compounds and alloys in heterogeneous catalysis, we explore the possibilities of using selected intermetallic compounds and alloy structures and phases as catalyst precursors to prepare highly active and CO_2_-selective methanol steam reforming (MSR) as well as dry reforming of methane (DRM) catalyst entities by controlled *in situ* decomposition and self-activation. The exemplary discussed examples (Cu_51_Zr_14_, CuZn, Pd_2_Zr, GaPd_2_, Cu_2_In, ZnPd, and InPd) show both the advantages and pitfalls of this approach and how the concept can be generalized to encompass a wider set of intermetallic compounds and alloy structures. Despite the common feature of all systems being the more or less pronounced decomposition of the intermetallic compound surface and bulk structure and the *in situ* formation of much more complex catalyst entities, differences arise due to the oxidation propensity and general thermodynamic stability of the chosen intermetallic compound/alloy and their constituents. The metastability and intrinsic reactivity of the evolving oxide polymorph introduced upon decomposition and the surface and bulk reactivity of carbon, governed by the nature of the metal/intermetallic compound-oxide interfacial sites, are of equal importance. Structural and chemical rearrangements, dictating the catalytic performance of the resulting entity, are present in the form of a complete destruction of the intermetallic compound bulk structure (Cu_51_Zr_14_) and the formation of an metal/oxide (Cu_51_Zr_14_, InPd) or intermetallic compound/oxide (ZnPd, Cu_2_In, CuZn) interface or the intertranformation of intermetallic compounds with varying composition (Pd_2_Zr) before the formation of Pd/ZrO_2_. In this Perspective, the prerequisites to obtain a leading theme for pronounced CO_2_ selectivity and high activity will be reviewed. Special focus will be put on raising awareness of the intrinsic properties of the discussed catalyst systems that need to be controlled to obtain catalytically prospective materials. The use of model systems to bridge the material’s gap in catalysis will also be highlighted for selected examples.

## 1 Introduction into the Scientific Concept

Intermetallic compounds represent an important and very fast growing group of materials in heterogeneous catalysis.^1−6^ Significant progress has been made over the past two decades with respect to synthesis, adsorption behavior, and the general understanding of bonding properties and structures. Several reviews covering almost all aspects of intermetallic compounds and alloys are currently available.^1−6^ With respect to catalytic applications, intermetallic compounds and alloys have continued to contribute significant progress to the understanding of a range of reactions, with the semihydrogenation of acetylene and methanol steam reforming at the forefront.^2^ Despite the large number of intermetallic compounds and alloys that are principally known (e.g., 6000 different only binary intermetallic compounds were known in 2014,^2^ a total number of 2500 publications with respect to the use of intermetallic compounds in catalysis have been published up to 2020^1^) and their widespread use in catalytic research, one key obstacle in their use clearly remains: even in the simplest reactions (and more valid for complex reactions, such as methanol steam reforming), the bulk and surface structure of the intermetallic compounds are generally not static, but increasingly dynamic.^1,2^ This renders the establishment of structure–property/activity/selectivity relationships not straightforward. The exemplary ZnPd intermetallic compound, which has particularly stirred up catalytic research in the past two decades, serves as a highly illustrative example in this respect. ZnPd is one of the most CO_2_-selective methanol steam reforming catalysts, and many aspects of its properties are already known.^7−11^ This structurally quite simple intermetallic compound is a prime example to show the highly dynamic nature of such materials in catalysis. ZnPd features structural alterations even if exposed to CO. Its catalytic performance in methanol steam reforming can be directly related to its structural instability and highly dynamic surface and bulk structure upon contact with the methanol and water reaction mixture.^2^ It is now widely accepted that the active phase is not the self-supported isolated intermetallic compound, but in fact an intermetallic compound-oxide ZnPd–ZnO interface, that is *in situ* formed during catalytic operation.^8^ A bifunctional operating mechanism is prevalent: ZnPd ensures methanol, and ZnO, water activation. Whether the interfacial region itself holds the active centers, or if spillover effects of activated species occur, is still subject to discussion. The features of ZnPd could be generalized to similar intermetallic compounds, where the structural dynamics appear unfortunate at first sight. However, this apparent disadvantage can be overturned if the structural alterations are controlled and the subsequent partial or complete decomposition of the intermetallic compound/alloy is steered in a catalytically meaningful way. As a consequence, intermetallic compounds and alloys would therefore be used only as highly defined precursor structures that are transformed—either by a selected pretreatment or in the reaction mixture itself—into the active/selective structure or phase.

This concept itself is not new: already in 1976, it was recognized that synthetic ammonia catalysts on the basis of intermetallic compounds consisting of a transition metal and a rare earth element, upon contact with the ammonia synthesis reaction mixture, give rise to decomposition and the formation of a metal–nitride interface.^12^ An array of such intermetallic compounds incorporating Co and Fe, e.g., HoFe_3_ (resulting in HoN and Fe) or CeCo_3_ (resulting in CeN and Co), yielded such interfaces. Similar observations were made for PrCo_2_, CeCo_2_, or PrCo_3_.^12^ Most remarkably, the authors particularly stated that only the *in situ* decomposed composite is active. The concept of using intermetallic compounds as precursor structures to generate more active materials has been extended to other reactions such as CO and CO_2_ methanation and hydrocarbon synthesis. For CO methanation, Coon et al. studied combinations of Ni, Co, Fe, and rare earth elements and found similar results (e.g., for LaNi_5_, ErNi_5_ or ErFe_3_, among others).^13−15^ More input has been provided by Craig et al. using actinide–transition metal intermetallic compounds for hydrocarbon synthesis. Despite their obvious niche application, the observations on ThNi_5_, ZrNi_5_, and UNi_5_ are of prime importance in the understanding of the operational principles of more recent and applicable intermetallic compounds.^13^*In situ* formation of Ni/ThO_2_, Ni/ZrO_2_, and Ni/UO_2_ upon exposure to the CO + H_2_ mixture was observed, and it was specifically stated that “...specific interfaces or specific interactions between metal and support [are observed]...”^15^ and that “...conventional Ni on ThO_2_ [prepared by impregnation] is less active than the Ni/ThO_2_ system obtained by ThNi_5_ decomposition...”^13,16^ The so-obtained mixture was identified as the active phase.^13,17^ For the Ni/ThO_2_ system, the increased H_2_S poisoning tolerance was attributed to a “bifunctional synergism,” resulting from the specifics of the element with which Ni was combined in the intermetallic compound precursor state.^15^ This already points to some kind of “memory effect,” indicating potential use for steering the catalytic properties of the resulting decomposition mixture. For ThNi_5_, it was stated that “nickel, formed as a decomposition product by the nature of the MNi_5_ compound, is probably the active species, but its properties are influenced by the nature of M in the MNi_5_ precursor state.”^15^

Decomposition of intermetallic compound catalysts for ammonia synthesis, CO oxidation, and selective hydrogenation, of Fe_91_Zr_9_^18^ and Pd_8_Si_19_,^19^ into (surface) Fe + ZrO_2–*x*_, as well as Pd + SiO_2_ was also observed. For the latter, the activity is due to a “very special surface distribution [of Pd and SiO_2_].”^20^ Recently, the concept of decomposing intermetallic compounds into an active state has also been extended to the methanol steam reforming performance of single-phase quasicrystals on especially an Al–Fe–Cu basis. The leaching behavior and the resulting formation of small copper particles has been determined to be strongly dependent on its individual composition.^21^

In recent years, the mostly unwanted, or at least not deliberately induced, decomposition of Pd- and Cu-based intermetallic compounds has given rise to especially CO_2_-selective methanol steam reforming catalysts.^7−11,22−35^ ZnPd, GaPd_2_, InPd, InPt, GaPt_2_, Cu_51_Zr_14_, or ZnNi, to name just a few, have one common structural denominator: resulting from partial or full *in situ* decomposition of an intermetallic compound precursor, the CO_2_-selective state is exclusively composed of an intermetallic compound (or metal)–oxide interface with shared activation and catalytic duties between the two constituting entities.^36^ Strong differences among the individual precursor materials with respect to adsorption, stability, or oxidation propensity have been observed, emphasizing the need for an approach less reliant on trial and error in order to induce and understand decomposition. Another reaction, where the concept of controlled intermetallic compound/alloy precursor decomposition is increasingly exploited, is the dry reforming of methane. Here, an additional level of complexity related to the carbon reactivity on mostly Pd–Zr systems is introduced,^37,38^ although the underlying principles of the concept are similar. A recent study on Ni–Y alloys also revealed in situ decomposition into Ni/Y_2_O_3_ composites with superior dry reforming activity.^39^ In a similar fashion, the stability of different Hf-based intermetallic compounds (e.g., NiHf or CoHf_2_) during dry reforming has been assessed.^40^

The same concept of creating supported-metal catalysts via decomposition of precursor structures was previously discussed for amorphous metal alloys (i.e., “metal glasses”). Several examples in the literature exist, which have demonstrated the potential to use such materials as promising catalyst precursors.^41,42^ In the present Perspective, we deliberately do not discuss such metal glasses but rather focus on prospective intermetallic compound precursors, which have the advantage of providing a highly defined starting structure. The corresponding alloy-related studies are essentially used to highlight the use of model systems to elucidate underlying mechanistic details of in situ decomposition, such as the reactivity of intermediary hydroxyl species resulting from water activation or reaction-induced carbon from methane activation.

As a consequence, the high structural dynamics giving in many cases rise to an at least partial decomposition is a matter of fact. However, destability of an intermetallic compound or alloy need not be a disadvantage per se. If a knowledge-based concept is established that allows the use of such materials to reproducibly and in a controlled way act as precursor structures for decomposition, access to more active and selective entities is granted.^43^ In the best way, nanocrystalline, highly stable supported intermetallic compound (or metal)–oxide composites of defined geometry, morphology, and electronic and thus, superior catalytic properties result. To accomplish this task successfully, the in-depth knowledge of factors and parameters influencing the decomposition is of the utmost importance. As will be clear from sections 2 and 3, for most of the examples these parameters (e.g., oxidation behavior, thermodynamic stability, modification, and catalytic performance of the resulting oxide or carbon reactivity) usually appear entangled. The term “knowledge-based concept or decomposition” is in the following used for an approach that takes advantage of the intrinsic properties of structurally, chemically, and electronically comparable intermetallic compounds/alloys to steer the decomposition without a widescale trial-and-error approach. Extending e.g., the ZnPd studies to GaPd_2_, CdPd, InPd, or ZnPt is such an example, which is documented by the similar valence band structure causing similar catalytic patterns in MSR.^1^

This Perspective introduces the widespread possibilities of using intermetallic compounds and alloys as precursor materials to prepare highly active and selective entities by controlled in situ decomposition and self-activation. We exemplify the advantages and possible pitfalls in using this approach by reviewing illustrative examples from our own expertise in section 2. Alongside the common feature of partial and/or full decomposition, the individual aspects of each discussed system will be assessed. Wherever possible, the discussed examples will be used to extrapolate the features to similar structures, thus, generalizing the concept. For each case study and material, a very short introduction into the state-of-the-art of the particular material in the chosen reaction will be given. The selection of the presented case studies is, on the one hand, driven by their use in two important reactions in the hydrogen economy and environmental science, methanol steam reforming and methane dry reforming. On the other hand, the selected materials are especially well-suited to show the scientific concept of this Perspective. The leading theme of the case studies with respect to methanol steam reforming is the importance of water activation and how this activation can be influenced by controlled decomposition. We selected two groups of intermetallic compounds/alloys: In section 2.1, two Cu-based materials, Cu_51_Zr_14_ and CuZn, are compared in their structural stability and methanol steam reforming performance, as the corresponding Cu/ZnO and/or Cu/ZrO_2_ systems have already displayed superior MSR properties. Section 2.2 is devoted to a direct comparison of the two Pd-based intermetallic compounds ZnPd and Ga_2_Pd. We link the (missing) structural instability of the respective intermetallic compounds directly to the MSR performance of the respective Pd/ZnO and Pd/Ga_2_O_3_ catalysts and the catalytic contribution of the reaction-induced oxide phase. The reactivity of reaction-induced carbon at Pd/ZrO_2_ interfaces resulting from in situ decomposition of Pd_2_Zr intermetallic compounds and Pd–Zr alloys is discussed in section 2.3. Section 3 deals with a set of key parameters that directly controls the catalytic performance outlined in section 2. The resulting metal-oxide phase boundary as the single most important parameter is discussed, alongside the consequences that arise in terms of oxide and carbon reactivity (sections 3.1 and 3.2). The combined knowledge of the intrinsic properties of both intermetallic compound/alloy structure and resulting decomposition products will then yield prerequisites to control the decomposition and obtain a leading theme to pronounced selectivity and activity. Control and steering of the decomposition is essentially possible by adjustment of the reaction environment (e.g., by changing the stoichiometry of the dry reforming reaction mixture to yield different interfacial carbon reactivities) and, therefore, its reduction/oxidation chemical potential. Another pathway of steering is related to varying the initial stoichiometry of the intermetallic compounds and alloys. We will show that, e.g., the water activation properties of stoichiometrically different Cu–Zn or Zn–Pd alloy samples is very much dependent on the initial stoichiometry.

## 2 Use of Controlled *in Situ* Decomposition of Intermetallic Compound and Alloy Precursor Structures to Create Highly Active and Selective Methanol Steam and Methane Dry Reforming Catalysts

### 2.1 Enhancing the Water Activation by *in Situ* Activation and Decomposition of Cu_51_Zr_14_ and CuZn Intermetallic Compounds and Alloys: Pathways to Metal–Oxide Systems with Superior Methanol Steam Reforming Performance

A first example to exploit the *in situ* decomposition of well-defined intermetallic compounds is the oxidative decomposition of Cu_51_Zr_14_ in a methanol steam reforming mixture into a very CO_2_-selective Cu/t-ZrO_2_ composite mixture.^23,28,29^ The addition (or substitution) of ZrO_2_ to already well-established Cu/ZnO catalysts allows an overcoming of the Cu sintering by structural stabilization of Cu by ZrO_2_. Direct interaction of Cu and the participating Zr species, including the formation of Cu–O–Zr bonds, has been suggested.^44−47^ The resulting Cu–ZrO_2_ interface has been suspected to host the active and selective sites. The contact of Cu metal to the tetragonal ZrO_2_ modification yields a particularly CO_2_-selective material.^42^ Structure-wise, the Zr–O phase diagram is a complex issue, as the thermodynamically more stable monoclinic polymorph has been previously reported to be of minor catalytic relevance for high CO_2_ selectivity.^45^ Tetragonal (or cubic) ZrO_2_ needs to be either externally stabilized by dopants (e.g., Y) or intrinsically stabilized by oxygen vacancies and/or particle size effects. Taking these features into account in the search for alternative preparation pathways to optimize the metal–oxide interfacial sites of CO_2_-selective Cu/ZrO_2_ methanol steam reforming catalysts, we end up with a difficult task.

**Figure 1:**
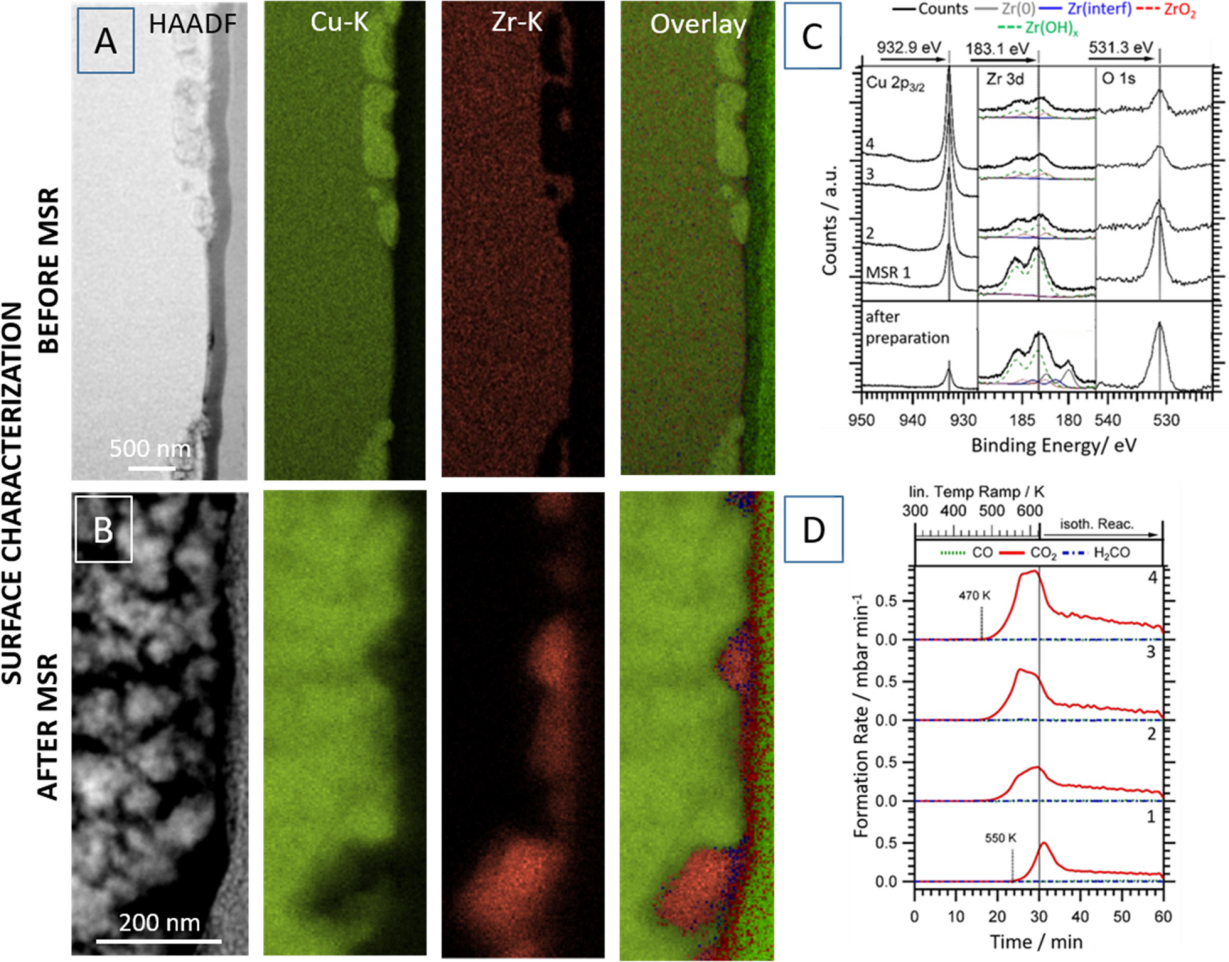
(Surface) structural, chemical, and catalytic characterization of the Cu_51_Zr_14_ intermetallic compound structure during methanol steam reforming up to 623 K. Panels A and B: STEM/EDX analysis of the surface-near regions of decomposed Cu_51_Zr_14_ before (A) and after one catalytic MSR cycle up to 623 K (B). The individual panels highlight the HAADF image and the Cu–K and Zr–K intensities. The overlay shows also the O–K intensity and to the right the Pt signal from the FIB sample preparation. Panel C: XPS surface chemical characterization before and after several MSR cycles. Panel D: Four consecutive catalytic methanol steam reforming profiles starting from the Cu_51_Zr_14_ intermetallic compound. Reproduced with permission from ref (28). Copyright 2021 Wiley-VCH.

**Figure 2:**
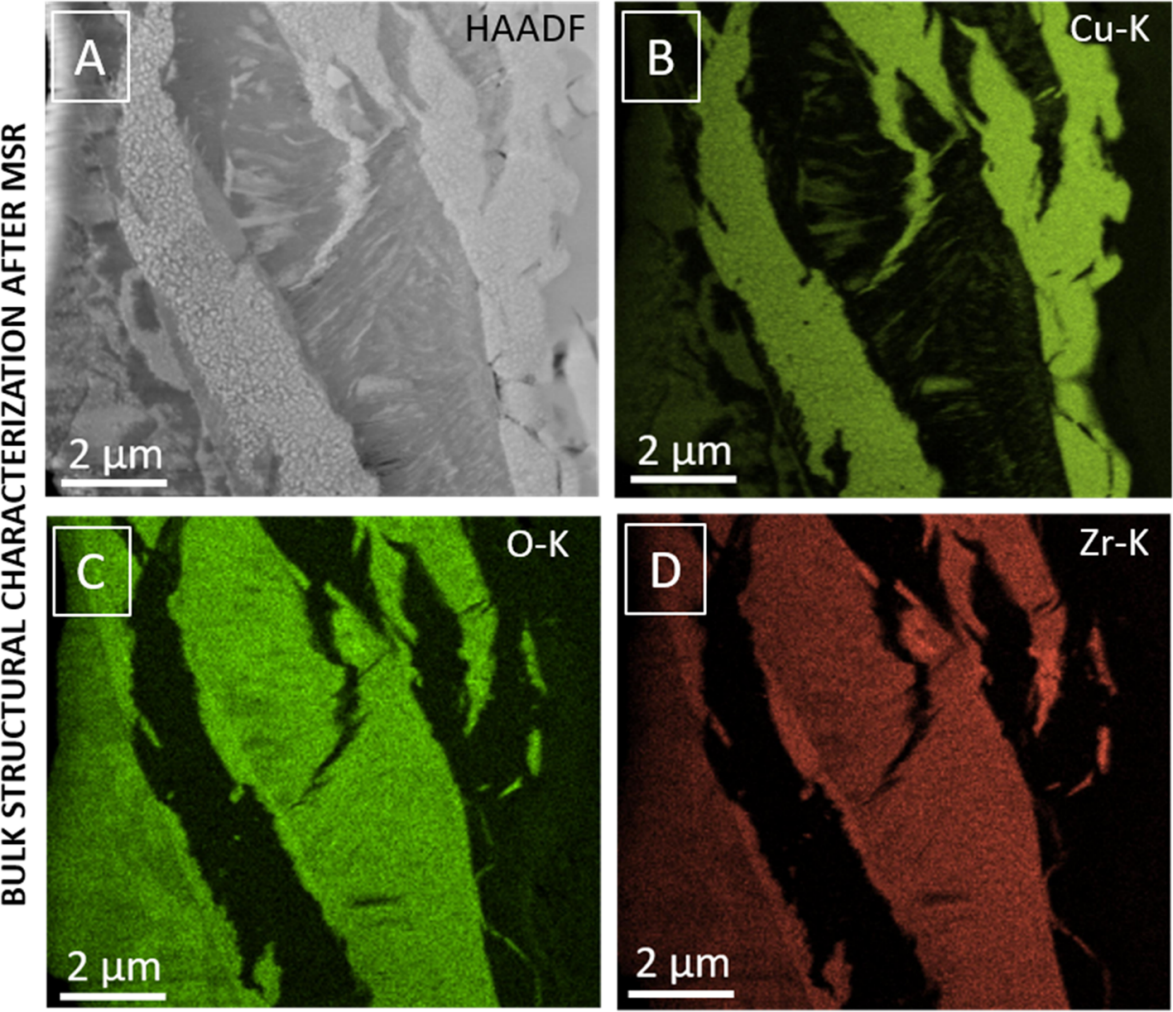
STEM/EDX characterization of the Cu_51_Zr_14_ intermetallic compound structure after a methanol steam reforming reaction up to 623 K. The individual panels highlight the HAADF image (panel A), the Cu–K (panel B), O–K (panel C), and Zr–K intensities (panel D). Reproduced with permission from refs (28) and (29).^32^ Copyright 2021 Wiley-VCH and American Chemical Society.

The use of Cu–Zr alloys or intermetallic compounds as precursor structures to access active and selective Cu/ZrO_2_ catalysts has been up to now limited to the exploitation of Pd- and Au-doped Cu–Zr metallic glasses.^48−51^ However, we experience limitations with respect to the ill-defined initial glassy state and the necessity for oxidative pretreatments to decompose the alloy before the methanol steam reforming experiment. The selection of a more defined Cu–Zr precursor intermetallic compound structure is a logical extension of the concept. Figures 1 and 2 highlight our results using a defined Cu_51_Zr_14_ intermetallic compound structure for oxidative decomposition in a methanol steam reforming mixture. We synthesized the intermetallic compound by reactive co-melting of metallic Cu and Zr, resulting in a quite uniform distribution of Cu and Zr within surface and bulk regions (Figure 1, panel A). Subjecting the Cu_51_Zr_14_ material to a methanol steam reforming reaction up to 623 K (Figure 1, panel B) yields decomposition into a Cu/ZrO_2_ composite consisting of surface-bound small Cu particles embedded in a ZrO_2_ matrix. Both X-ray and electron diffraction confirm the almost exclusive presence of tetragonal ZrO_2_. In essence, the *in situ* decomposition during methanol steam reforming yields the anticipated Cu/t-ZrO_2_ sample with an extended—due to the intimate contact between Cu in the ZrO_2_ matrix—interface between Cu and tetragonal ZrO_2_. Destruction of Cu_51_Zr_14_ affects the surface and bulk in a similar way (Figure 2).^23,28,29^

Revisiting the prerequisites for CO_2_-selective MSR, we note that the key criterion is efficient methanol and water at dedicated (interfacial) sites. The role of Cu is still controversially discussed, with both purely geometric strain and ionic effects (Cu vs. Cu^+^ species) put forward.^28^ However, water activation remains crucial. Numerous studies on different intermetallic compounds reveal the role of the “support” formed by decomposition of the catalytic precursor^7−11,22−35^ (ZrO_2_ in this case), going well beyond simple stabilization of the distribution of reactive Cu particles. Reversible hydroxylation of ZrO_2_ (or special interfacial sites), invoking a bifunctional synergism with shared duties between Cu (methanol activation) and ZrO_2_ (water activation), is very important. Tetragonal ZrO_2_ arising from *in situ* decomposition is indeed capable of efficient reversible formation of surface Zr–OH species, as documented by the Zr–OH component in Zr 3d XP spectra after each of the four consecutive MSR runs (Figure 1, panel C). This directly translates into a very CO_2_-selective Cu/tetragonal ZrO_2_ material (Figure 1, panel D). Ongoing *in situ* activation of Cu_51_Zr_14_ during the four displayed MSR cycles occurs, as judged by the shift of the CO_2_ light off temperatures to lower values after each consecutive run. The activity of the observed material is 10^2^ times higher than similar Cu/ZrO_2_ systems described in the literature and still exceeds a conventional Cu/ZnO catalyst by a factor of 3 (studied under identical conditions).^28^

We identify essentially two factors steering the decomposition of Cu_51_Zr_14_ and the exclusive formation of Cu/tetragonal ZrO_2_. First, we note the high oxidation propensity of Zr and the associated high formation enthalpy of ZrO_2_^52^ formed by Cu_51_Zr_14_ decomposition. Also, our own model system studies on differently preprared Cu-ZrO_2_ materials starting from different alloy precursors have shown that keeping Zr in its metallic state during preparation is extremely difficult. However, the inevitable oxidation of Zr and the formation of Zr^0^/Zr^4+^ entities in fact provide an efficient approach to Cu–Zr^0^/Zr^4+^ materials that can be deliberately switched between a CO-, HCHO-, and CO_2_-selective state by the preparation process.^53,54^ The key criterion is the different hydroxylation ability of the different Zr species formed during synthesis. A second (geometric) steering factor is the particular epitaxial stabilization of the Cu metal–tetragonal ZrO_2_ interface.^29^ The observed almost perfect epitaxial match is particularly aided by the well-defined precursor Cu_51_Zr_14_ structure.

Summarizing, the Cu_51_Zr_14_ case study provides a perfect example of how the decomposition of a highly defined precursor state yields an outstandingly CO_2_-selective methanol steam reforming catalyst, with a bifunctional operating mechanism being directly deducible. The discussed concept is also valid for other nominal initial Cu/Zr stoichiometries. Variation of the Cu/Zr ratio from 9:2 over 2:1 to 1:2 (essentially starting from Cu_5_Zr or CuZr_2_ structures with Cu metal and Zr metal by-components) and in situ decomposition during MSR essentially yields similar catalytic patterns with respect to CO_2_ selectivity and hydroxylation propensity of the participating Zr^4+^ species.^23^

The results from the Cu_51_Zr_14_ case study can be perfectly generalized to investigations of the hydrogen production following CO_2_-selective methanol steam reforming on CuZn alloy precursor states. Mainly driven by the in-depth understanding of technical Cu/ZnO methanol synthesis catalysts, the importance of the Cu^0^/ZnO interface in both preparation and activation has been repeatedly stressed.^55^ The addition of Zn has been previously suspected to lead to a clear improvement of catalytic properties, despite the fact that the exact role of Zn had not been clarified until recently. In particular, a plethora of essentially contradictory interpretations of the role of the Cu/ZnO interface have been put forward, including a “Cu–Zn” alloy model, with the suspected formation of a Cu–Zn(OH) species during MSR.^55^

The model concept using a UHV-based methodological approach is particularly feasible here, as the eventual segregation behavior of Zn and the associated redox chemistry of both Cu and ZnO (and their interface) are more easily followed. To optimize the CO_2_ selectivity, we scrutinized a series of brass samples with different nominal stoichiometries (CuZn37, CuZn10, and CuZn15) and a near-surface Cu–Zn alloy state, accessed through thermal Zn deposition and subsequent annealing treatments. Evaporation of between 5 and 12 monolayers of Zn onto a Cu foil at 300 K, followed by a short thermal annealing step (10 min) at 523 K, induces the formation of a CuZn ∼10:1 near-surface alloy state with superior MSR properties.

**Figure 3:**
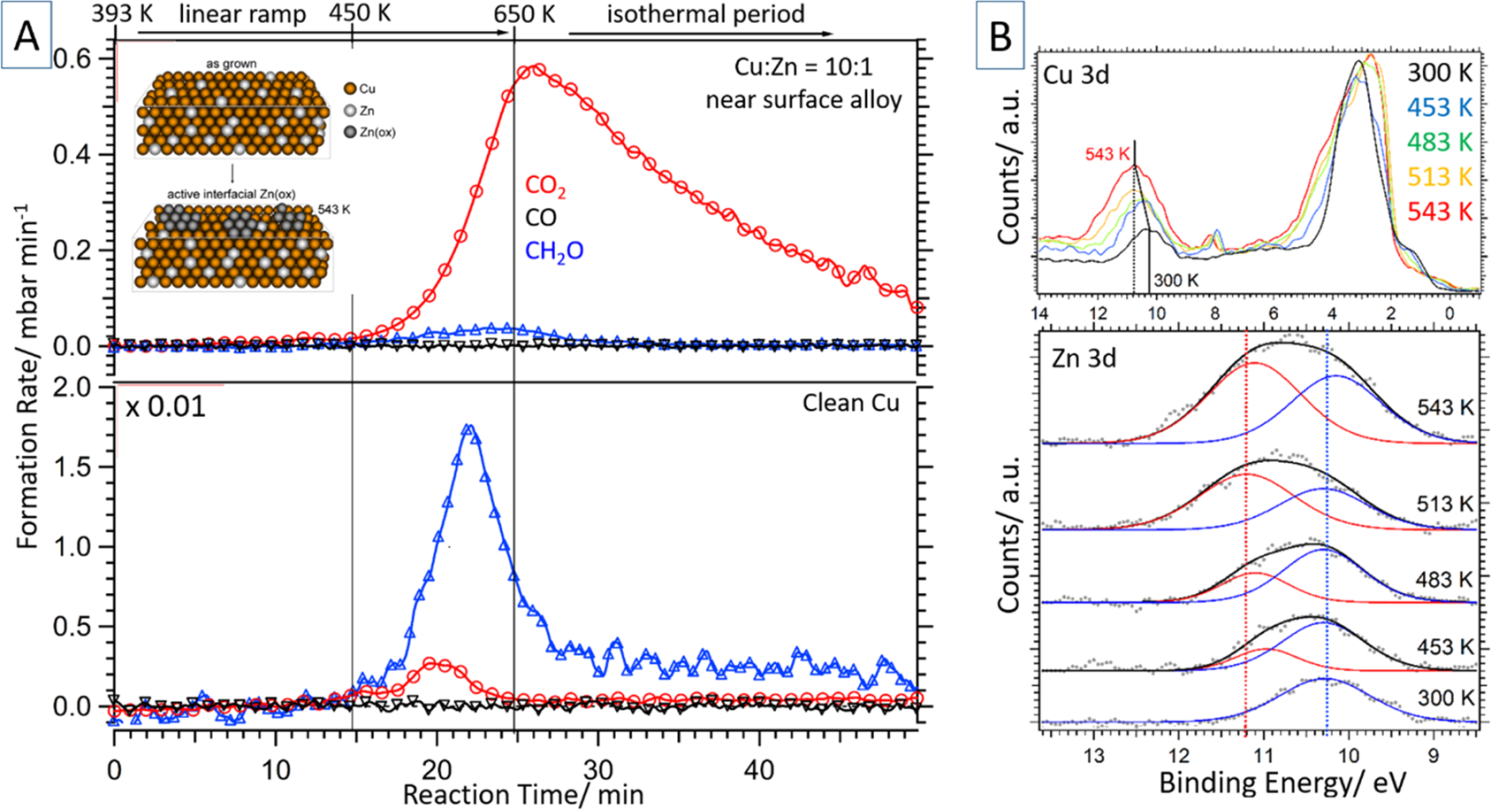
Panel A: Catalytic methanol steam reforming profiles on clean Cu (lower panel) and a Cu:Zn = 10:1 near surface alloy (top panel). A schematic of the formation of the active centers during MSR is shown as inset. Panel B: *In situ* X-ray photoelectron spectra (top, Cu 3d; bottom, Zn 3d region) collected on an initial Cu/Zn = 10:1 near surface alloy at 130 eV during methanol steam reforming (0.12 mbar methanol + 0.24 mbar water). The Zn 3d region is deconvoluted into bimetallic CuZn (blue, 10.25 eV) and oxidic Zn (red, 11.2 eV) components. Reproduced with permission from ref (55). Copyright 2021 Wiley-VCH.

The MSR profile (Figure 3A) of this alloy state in relation to pure Cu reveals that methanol is fast converted with water at almost 100% CO_2_ selectivity between 530 and 623 K. The *in situ* near-ambient pressure XP spectra collected during catalytic MSR operation (Figure 2B) point out that this CO_2_-selectivity goes along with a transition from a purely bimetallic Zn 3d component at 300 K to an almost 1:1 mixture of oxidic and bimetallic Zn at 543 K. The start of the Zn segregation to the surface can be pinpointed to ∼450 K, as indicated by a binding energy shift of the Zn 3d peak and a rise in the Zn 3d/Cu 3d peak ratio. Summarized in the inset in Figure 3A, the CuZn ∼10:1 near-surface alloy precursor state provides the optimum Zn loading and distribution for an Cu/Zn_ox_ interface with a high number of active sites providing high CO_2_ selectivity. A bifunctional synergism prevails, with Cu providing fast methanol dehydrogenation to formaldehyde, while Zn_ox_ sites are essentially responsible for water activation. Missing Zn_ox_ sites, as well as the presence of a fully Cu blocking passivating Zn_ox_ layer lead to catalyst deactivation.

The obtained results already point out an inherent problem of a delicate stoichiometric balance of Cu and Zn in the precursor state to obtain a CO_2_ selective state during MSR. To underline this importance, in the present case study all brass samples and all too Zn-rich near-surface alloys exhibited formation of such a Zn_ox_ passivating layer. Exact tuning of the initial level of Zn doping is imperative to, e.g., promoting formate reactivity at an optimized Cu/Zn_ox_ interface. Both CO_2_ selectivity and MSR activity directly scale with the extent of the Cu/Zn_ox_ interface, which is a result of the optimum precursor stoichiometry and the subsequent Zn segregation (and oxidation) to the surface during *in situ* activation.

### 2.2 Teamwork or Not? Enhancing the Methanol Steam Reforming Performance by Bifunctionally Operating *in Situ* Activated Intermetallic Compound–Oxide Interfaces: ZnPd vs GaPd_2_

The group of intermetallic compounds based on 8–10 group metals was initially introduced by Iwasa et al. in the mid 1990s, mostly to overcome the poor sintering stability and associated deactivation of conventional Cu/Zn/Al_2_O_3_ catalysts.^10^ Starting with Pd and Pt particles on selected oxides, reduction in hydrogen at temperatures up to 773 K (depending on the specific oxide) yielded small intermetallic compound particles supported on a more or less reduced oxide support. Depending on the oxide, very different catalytic patterns in methanol steam reforming resulted. Depositing Pd or Pt on, e.g., SiO_2_, an oxide usually considered hard to reduce, preserves the metallic state upon reduction and only methanol dehydrogenation is observed (intermetallic compound formation starting from Pd or Pt on SiO_2_ (or Al_2_O_3_) can be triggered upon reduction in hydrogen, but needs much higher temperatures (*T* ≥ 873 K)^56^). In contrast, deposition of small Pd or Pt particles on ZnO, Ga_2_O_3_, or In_2_O_3_ and subsequent hydrogen reduction yields the highly CO_2_-selective intermetallic compounds ZnPd, GaPd_2_, and InPd.^7−11,24−27,30−35^ These early observations have triggered extensive studies to unravel the full mechanistic details of MSR operation. Extension to structurally similar compounds, such as CdPd, revealed the common electronic valence band structure of metallic Cu, ZnPd, GaPd_2_, InPd, and CdPd (among others) to be the crucial catalytic steering parameter.^43^ In due course, first attempts were made to separate the structural and catalytic contributions of intermetallic compounds and oxides. Several key observations, in turn fitting to a larger picture of *in situ* activation of intermetallic compounds and the establishment of structure-selectivity correlations, were made: (i) As discussed for Cu–Zn,^55^ the stoichiometric balance between both constituents of the intermetallic compounds was found to be a generally important parameter to establish a highly CO_2_-selective material. This was by far best studied on the archetypical ZnPd compound.^7−11^ ZnPd exhibits a rather large compositional range, where a large deviation from the ideal stoichiometry can be structurally tolerated.^9^ However, these compositional deviations go at the cost of a different electronic structure, formation of passivating oxide layers, and composition-dependent catalytic patterns.^43^ Largely neglected for a long time were in fact the catalytic and structural properties of the supporting oxide.^10,43^ Different catalytic profiles were obtained using either ZnO, Ga_2_O_3_, or In_2_O_3_ as supports.^7−11,24,25,27,33^ ZnO and In_2_O_3_ are both highly CO_2_-selective methanol steam reforming catalysts,^11,57^ but Ga_2_O_3_ itself features a vital formate- and oxygen vacancy-mediated (reverse) water–gas shift reactivity, spoiling the CO_2_ selectivity.^58,59^ In comparison to In_2_O_3_, ZnO and Ga_2_O_3_ are hard-to-reduce oxides. In_2_O_3_ readily loses lattice upon annealing in either pure hydrogen or during MSR, and as such, most catalytically relevant properties are oxygen vacancy-dominated.^60,61^ As a consequence of both points discussed above, the in situ stability and eventual decomposition into an (inter)metallic compound/metal-oxide system and its dependence on composition has drawn particular attention. Naturally, this again raises the question of the influence of the metal-oxide phase boundary on the catalytic properties.

**Figure 4:**
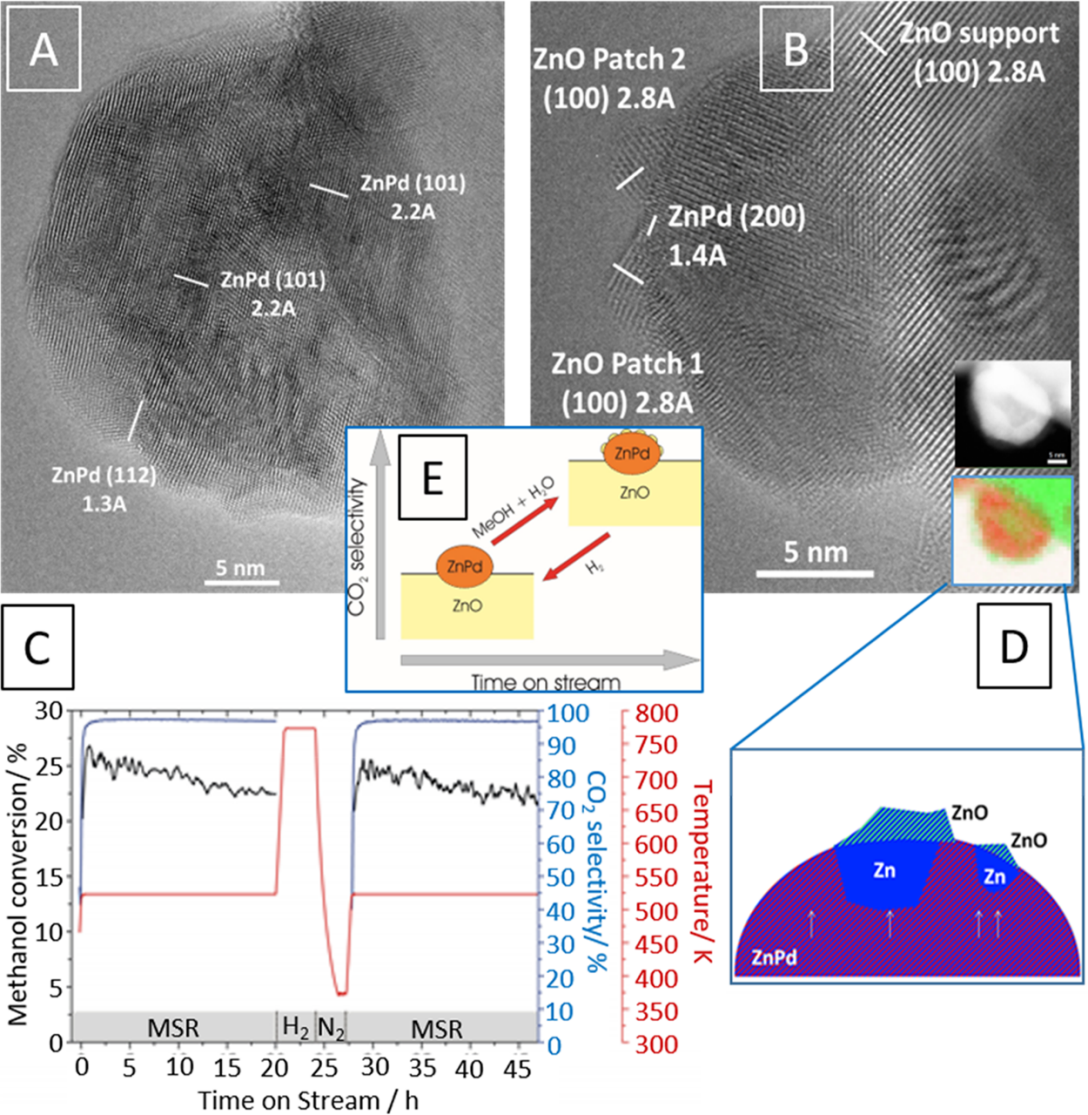
High-resolution electron microscopy images of a ZnPd/ZnO catalyst before CO_2_ selectivity is observed (panel A) and in the CO_2_-selective state (panel B). The catalytic methanol steam reforming experiment is highlighted in panel C. Panel D schematically depicts the CO_2_-selective ZnPd/ZnO interface in situ formed during catalytic MSR operation as derived from high-resolution and EELS imaging (inset in panel B). Panel E shows an overview of the CO_2_ selectivity as a function of structural transformation of the catalyst. Reproduced with permission from ref (8). Copyright 2021 Wiley-VCH.

The importance of this (*in situ* formed) boundary is best appreciated if the MSR performance of isolated ZnPd^8^ and GaPd_2_^30^ is compared. Figure 4, panels A and B showcase a direct high-resolution TEM comparison of ZnPd after initial contact with the reaction mixture at 573 K (panel A) and in the most CO_2_-selective state (panel B). Following the initial explanation of assigning the full catalytic action to ZnPd alone,^10,43^ the catalyst state displayed in panel A would be essentially CO_2_-selective, as it apparently consists of small ZnPd particles supported on reduced ZnO. The MSR profile (panel C), however, features an induction period of 30–60 min before the CO_2_ selectivity strongly increases. Thus, the catalyst *in situ* self-activates and transforms itself into the state of panel B. The critical structural difference between the two states is the appearance of ZnO patches on the surface of the ZnPd particle. This ZnO arises from oxidative *in situ* decomposition of Zn-rich areas within the chemically extremely inhomogeneous ZnPd particles (inset in panel B and panel D) and is structurally and electronically very different from the ZnO support.^62^ It is a direct consequence of *in situ* activation. The sequence of transforming the initial impregnated Pd/ZnO catalyst into the CO_2_-selective state involves (i) a reduction in hydrogen to form the ZnPd/ZnO state (which is per se not CO_2_-selective) and *in situ* formation of ZnPd(ZnO)/ZnO, which represents the CO_2_-selective state. The formation of the ZnO patches does not occur via a classic “strong metal-support interaction” effect but is purely a result of a chemical reaction of the Zn-rich areas with the MSR reaction mixture.^62^

**Figure 5:**
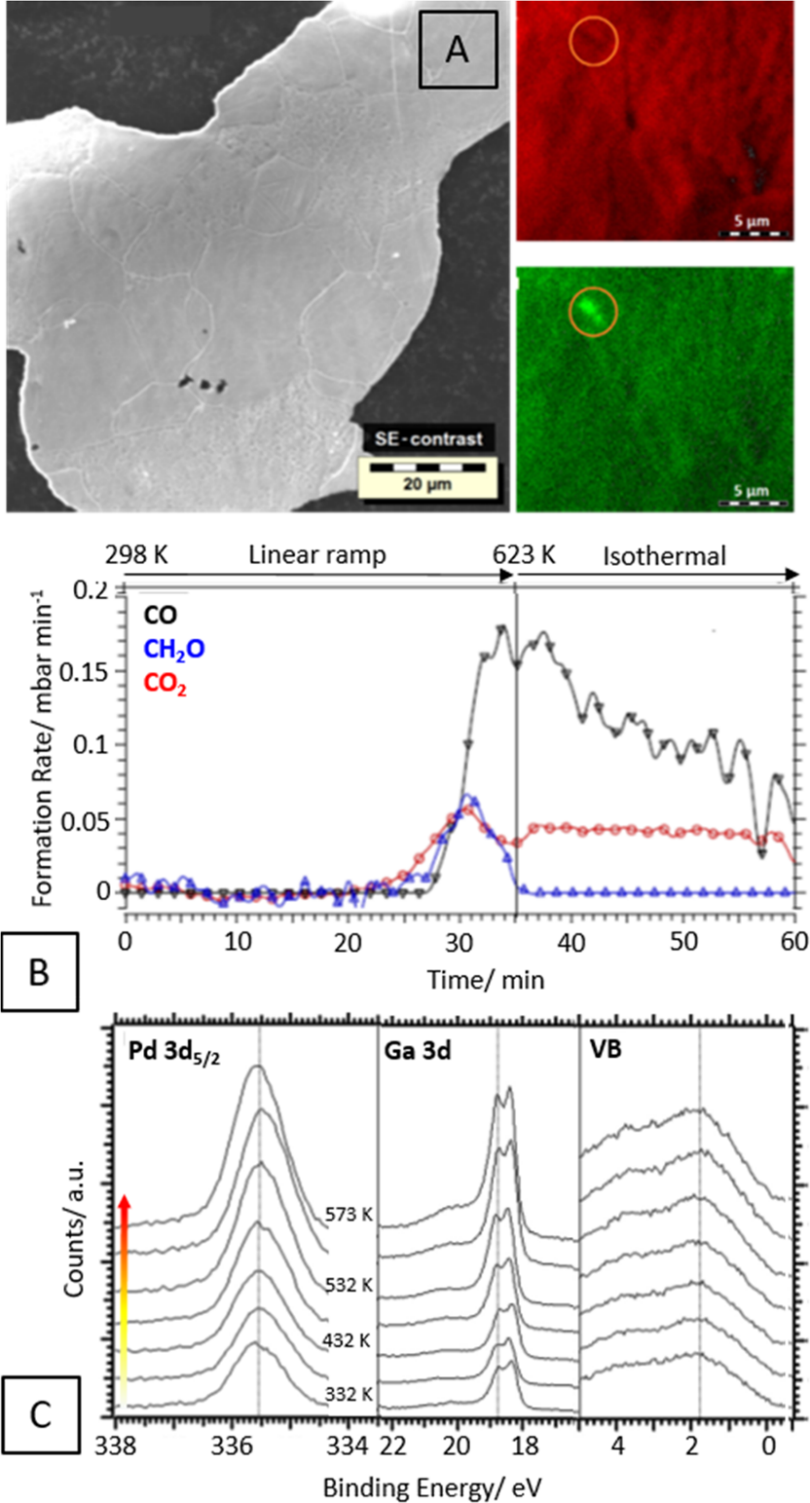
Panel A: SEM/EDX analysis of the isolated self-supported bulk GaPd_2_ intermetallic compound with the elemental Pd (Pd-M, green) and Ga (Ga-L, red) distribution as determined by EDX. Panel B: Catalytic methanol steam reforming profiles (12 mbar methanol + 24 mbar water). Experimental details given in ref (22). Panel C: *In situ* collected Pd 3d_5/2_(left), Ga 3d (middle), and valence band (right) XP spectra collected during methanol steam reforming (12 mbar methanol + 24 mbar water) on GaPd_2_. For maximum surface sensitivity, the Pd 3d_5/2_ signal was measured at 470 eV photon energy and the Ga 3d and valence band signals at 170 eV. The arrow in panel C indicates the increasing temperature from 332 to 573 K. Reproduced with permission from ref (24). Copyright 2021 Elsevier.

The importance of the *in situ* activation of intermetallic compounds during MSR to deliver CO_2_-selective materials is further strengthened by similar experiments starting from isolated oxide-free GaPd_2_. In theory, this particular material is expected to behave similarly to ZnPd on the basis of reports on the MSR performance of Pd/Ga_2_O_3_ catalysts.^22,24,33,63^ As for Pd/Ga_2_O_3_, reduction yields a CO_2_-selective Ga_2_O_3_-supported GaPd_2_ intermetallic compound. To answer the question whether the isolated GaPd_2_ is equally prone to self-activation, a self-supporting bulk-like GaPd_2_ film was prepared by alternating deposition of Pd and Ga layers and subsequent thermal annealing (Figure 5, panel A). The catalytic MSR profile, however, indicates no CO_2_-selective state (Figure 5, panel B).^22^ CO is the main product due to the dominating methanol dehydrogenation with both CO_2_ and formaldehyde only formed as minor byproducts. The reason for this behavior is clear from the *in situ* collected XP spectra during MSR operation (Figure 5, panel C): no oxidic Ga_2_O_3_ component arises during MSR operation, pointing toward missing self-activation. Only if the Ga_2_O_3_ support is present from the beginning can a selectively functioning entity with shared duties between GaPd_2_ (methanol activation) and Ga_2_O_3_ (water activation) arise.^22,24,63^

**Figure 6:**
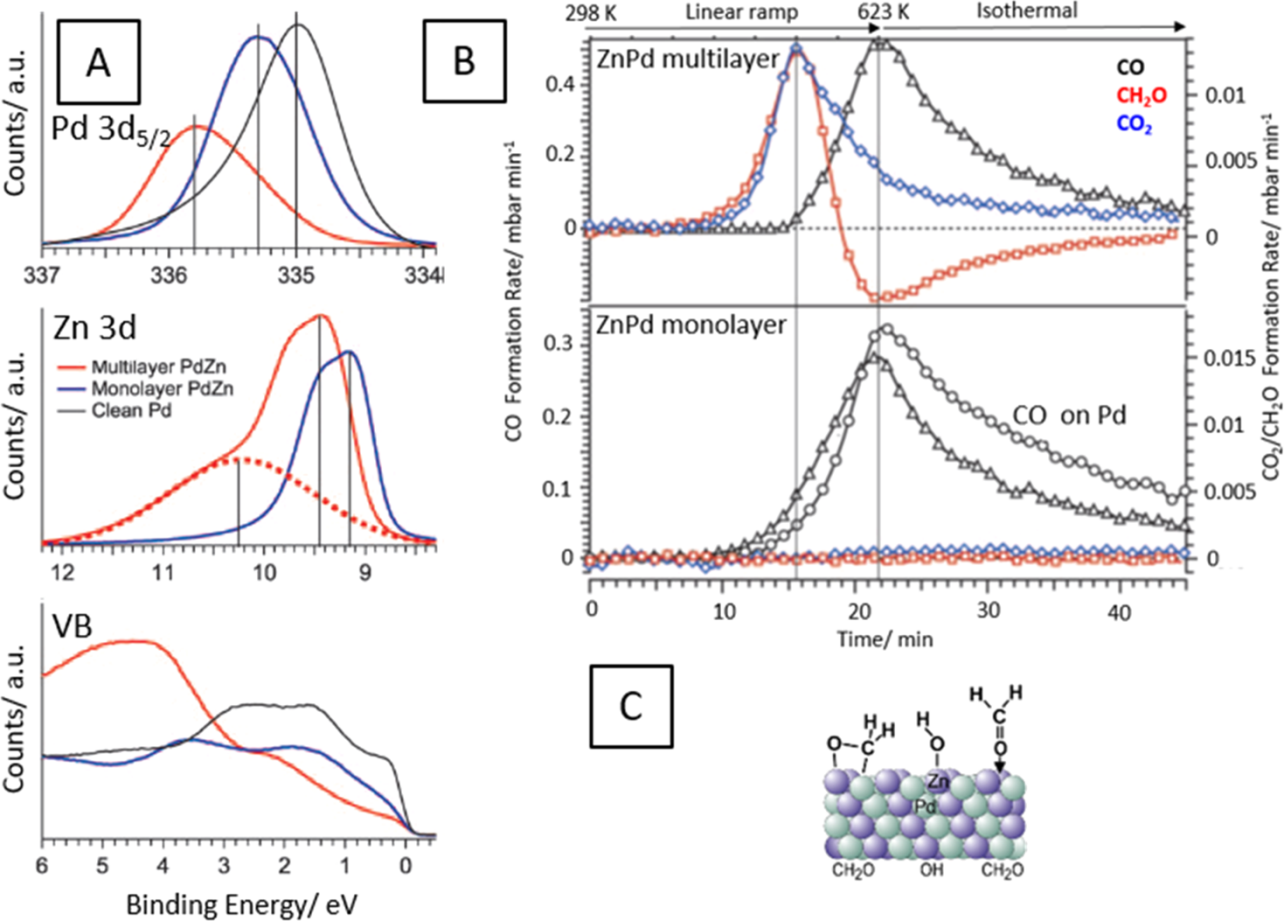
Panel A: *In situ* XP spectra (Pd 3d_5/2_, Zn 3d and valence band regions) collected during MSR on a 1:1 ZnPd multilayer (red spectra) and on a respective ZnPd monolayer (blue spectra). Black spectra: metallic Pd reference. The *in situ* formed, oxidized ZnOH component is shown as a broken red line in the middle panel. For maximum surface sensitivity, the Pd 3d_5/2_ signal has been measured at 650 eV photon energy, the Zn 3d and valence band signals at 120 eV. Reaction conditions: 0.12 mbar methanol + 24 mbar water at 553 K. Panel B: MSR profiles on the multilayer PdZn 1:1 alloy (upper panel) vs MSR reaction on a monolayer PdZn surface and MSR reaction on clean Pd foil (lower panel). Reaction conditions: 12 mbar methanol + 24 mbar water. Experimental details given in ref (35). Panel C: Side view of the multilayer PdZn alloy with possible surface intermediates en route toward CO_2_. Reproduced with permission from ref (35). Copyright 2021 Wiley-VCH.

The outstanding role of ZnPd with respect to self-activation is confirmed by dedicated model catalyst studies utilizing differently prepared ZnPd materials (Figure 6) and also provides the link to the CuZn experiments discussed in section 2.1.^35^ The electronic structure of a thin ZnPd monolayer alloy very much resembles the one of pure metallic Pd. A missing oxidized Zn component in the respective in situ collected XP spectra (panel A) explains the suppressed water activation and full methanol dehydrogenation to CO (panel B, lower panel). In contrast, a bulk-like ZnPd alloy features both the electronic valence band structure of Cu and an oxidized Zn component. The latter arises from *in situ* self-activation—similarly as discussed for the powder ZnPd/ZnO material—and gives rise to a CO_2_-selective material in MSR (panel B, upper panel). The bifunctional operating mechanism enabling methanol and water activation on different sites of the bulk ZnPd sample is schematically depicted in panel C.

### 2.3 Steering the Methane Dry Reforming Activity of Pd–Zr Intermetallic Compounds and Alloys by Controlled *in Situ* Decomposition Yielding Pd-ZrO_2_ Interfaces with Beneficial Carbon Reactivity

The dry reforming of methane (DRM) reaction is considered a promising method to convert two harmful climate-harming gases, CO_2_ and CH_4_, into useful syngas, which can be further used to access a range of useful synthetic fuels. It is possible to steer the follow-up reactions by adjusting the H_2_/CO ratio in the produced syngas mixture. 1:1 ratios allow carbonylation or hydroformylation processes, while the synthesis of renewable fuels requires H_2_/CO ratios higher than 2.^64,65^ Application-wise, the coking issues, especially on the widely used Ni-based materials, represent the most serious obstacle.^66−69^ Early attempts to improve the Ni coking resistance yielded promising bimetallic NiPd DRM catalysts supported on ZrO_2_.^70^ On a mechanistic level, both ensemble and ligand effects at the bimetallic surface can account for the methane-activating role of the intermetallic components, but the duty of the intermetallic compound (or alloy)–oxide interface is less clear. For inert supports, an eventual cocatalytic role of the metal–oxide interface is apparently less pronounced in the presence of a material that may activate both CO_2_ and CH_4_, such as pure Ni. Steering the level of bifunctional operation is possible by mixing Ni with an oustanding CH_4_ activator with at the same time inferior CO_2_ activation properties in its pure state, such as Pd. Consequently, the associated promotion of CO_2_ activation on Pd requires a comparatively higher number of Pd–oxide interfacial sites.^37,38^

This lays out the general strategy to employ intermetallic compounds and alloys in the knowledge-driven development of active methane dry reforming catalysts: we should focus on the preparation of the most extended (inter)metal(lic)–oxide interface providing superior methane activation on the *in situ* activated intermetallic compound or metal component and enhanced CO_2_ activation properties of the oxide component. Both oxygen vacancy-mediated (as a consequence of surface reducibility) and surface-chemistry mediated (as a consequence of basic surface sites enabling CO_2_ activation as reactive carbonate intermediates) parameters are considered central for high DRM activity. With respect to the use of intermetallic or alloy precursor structures, bulk intermetallic samples are particularly suited to trigger partial or quantitative decomposition into metal–oxide systems with a large contact area. This can in principle be achieved by precatalytic treatments, such as reductive activation or following special leaching techniques, or achieved through direct *in situ* activation in the reaction mixture. We have shown in the preceding sections that this is a particularly worthwhile approach for Pd–Zr and Cu–Zr systems in methanol steam reforming to access a large amount of phase boundary sites.^23,28,29,37,38^

**Figure 7:**
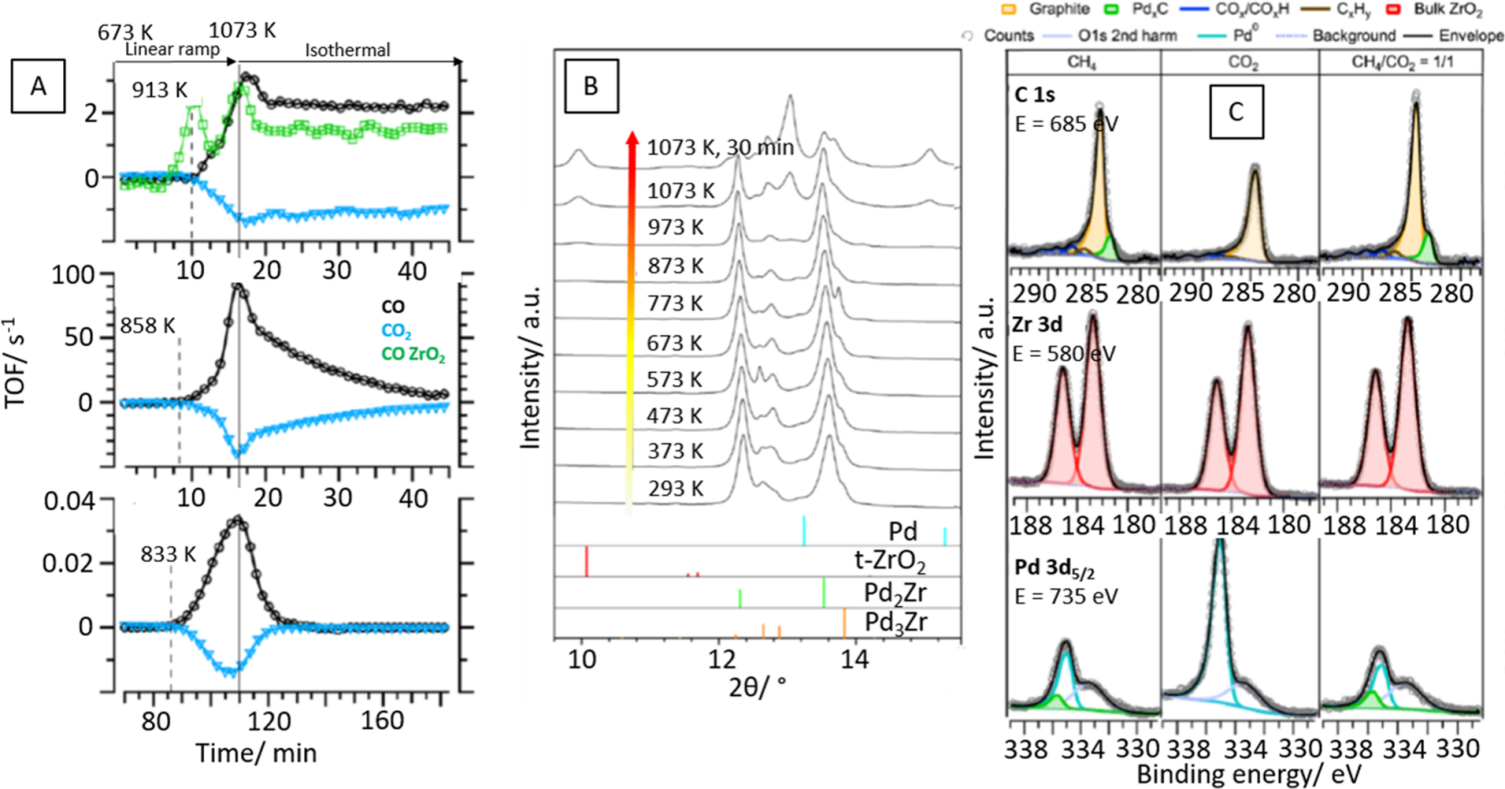
Panel A: DRM profiles on the CVD-prepared subsurface Zr^0^–Pd foil precatalyst vs a single-ZrO_2_ film (upper panel), on the Pd_2_Zr bulk-intermetallic precatalyst (middle panel), and on the supported Pd–ZrO_2_ powder reference catalyst. Detailed reaction conditions given in refs (37) and (38). Panel B: Synchrotron-based *in situ* X-ray diffractograms of the bulk-intermetallic Pd_2_Zr catalyst collected in a CH_4_/CO_2_ (ratio 1:1) reaction mixture between 293 and 1073 K. Gas flow: 2 mL min^–1^ at ambient pressure with a heating rate of 20 K min^–1^. The colored bars mark the positions of the respective reference reflections. Panel C: High-resolution *in situ* XP spectra of the C 1s, Zr 3d, and Pd 3d_5/2_ recorded at 973 K on the Pd_2_Zr precatalyst (excitation energies chosen for 400 eV photoelectron kinetic energy). Left spectra, 0.3 mbar pure CH_4_; middle spectra, 0.3 mbar pure CO_2_; right spectra, 0.15 mbar CH_4_ + 0.15 mbar CO_2_. TOF values obtained by normalization of the molar rates to the geometrically estimated total number of surface Pd atoms. Details of calculations given in refs (37) and (38). Reproduced with permission from refs (37) and (38). Copyright 2021 Wiley-VCH and MDPI.

The importance of the quality of the evolving Pd-ZrO_2_ phase boundary sites with respect to activation properties and the associated carbon reactivity evolving from *in situ* decomposition of different Pd–Zr intermetallic compounds and defined alloys is summarized in Figure 7. A comparative catalytic DRM characterization of a subsurface Zr^0^-doped Pd sample (representing a near-surface model alloy catalyst), a bulk Pd_2_Zr intermetallic compound, and a Pd/ZrO_2_ reference catalyst already reveals different active states (panel A). The structural denominator of the intermetallic compound/alloy sample is the decomposition into Pd/ZrO_2_ during a DRM treatment (monitored by *in situ* X-ray diffraction during DRM operation up to 1073 K, panel B) exceeding the activity of the impregnated Pd/ZrO_2_ catalyst by a factor of 100. The high activity is directly linked to the fast reaction of highly reactive Pd carbide species (i.e., dissolved carbon species inside the Pd^0^ bulk) toward CO at the Pd-ZrO_2_ phase boundary, providing the necessary efficient CO_2_ activation sites. This carbide species is visible in the corresponding in situ collected XP spectra of both the C 1s and the Pd 3d region (panel C). This obviously crucial component is missing for the subsurface Pd–Zr alloy, which forms extended ZrO_2_ islands on top of a quasi-infinite Pd bulk, serving as a sink for carbon.^37,38^ Consequently, the transport of reactive carbon to the interface is suppressed, deactivating the associated Pd–ZrO_2_ interface in comparison to the bulk Pd_2_Zr sample.^37,38^ This observation is similar to the ones made for the ThNi_5_ materials discussed in the context of hydrocarbon synthesis.^15^ The crucial role of the carbon reactivity will focused upon in section 3.2 in more detail.

## 3 Phase Boundary Effects to Prepare Selective and Active Materials Following *in Situ* Decomposition of Intermetallic Compound/Alloy Precursors

The present section seeks to identify key factors determining the pathway of structural decomposition en route to active and selective catalysis for the materials outlined in the case studies. We restrict ourselves here to the methanol steam reforming and methane dry reforming performance, but the concepts can be extended to similar systems at will. An obvious prerequisite is the existence of an intermetallic compound or at least an alloy, which is directly linked to the formation of metal–metal bonds or the (partial) solubility of at least two metals. Subsequently, the thermodynamic stability limits of the intermetallic compounds/alloys need to be approached under the chosen reaction conditions. As such, these conditions are not static in the course of the reaction and may switch between reductive and oxidative. For methanol steam reforming, the reaction conditions change from oxidative in the beginning to increasingly reductive as the reaction progresses and more hydrogen is formed. To obtain a highly dispersed metal–oxide system via intermetallic compound/alloy *in situ* decomposition, a high oxidation propensity of one part (in case of a binary intermetallic compound) of the catalyst material is imperative. Hence, the combination of a noble metal with an easily passivating metal is usually a promising starting point. As a conclusion, we will use the knowledge derived from the identified key factors to propose promising candidates of intermetallic compounds, whose testing might result in catalytically prospective materials

The stability of the *in situ* formed active and selective metal-oxide phase boundary is the single most important parameter, determining the catalytic properties of the entire catalyst material. It is connected not only to the stability of the intermetallic compound or alloy precursor structure, steering the structure, morphology, and electronic properties of the resulting metal-oxide phase boundary, but directly influences the physicochemical properties of the phase boundary itself. Two of these properties are discussed in the next section: the reactivity of the resulting oxide polymorph and the reactivity of reaction-induced carbon.

**Figure 8:**
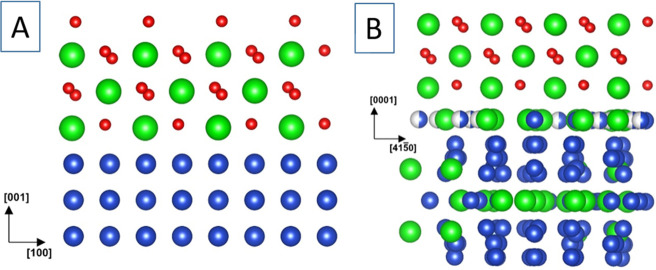
Ball models of the epitaxial Cu/t-ZrO_2_ (panel A) and Cu_51_Zr_14_/t-ZrO_2_ (panel B) relationships. Side view of Cu(001)//tetragonal ZrO_2_ (112) and Cu_51_Zr_14_(0001)//tetragonal ZrO_2_ (112). Color code: Zr, green; O, red; Cu, blue. Reproduced with permission from ref (29). Copyright 2021 American Chemical Society.

In this section, we focus on one key parameter, featuring two sides of the same coin and serving as a prime example to show its entangled nature. As discussed for the CO_2_-selective state of Cu/ZrO_2_ catalysts, the interface of Cu to tetragonal ZrO_2_ particularly stands out in high CO_2_ selectivity. Apart from beneficial surface chemical issues of tetragonal ZrO_2_, we have shown that the interface between Cu and tetragonal ZrO_2_ is particularly stabilized by epitaxial effects.^29^ The reported lattic mismatch between the tetragonal ZrO_2_ (012) and cubic Cu (111), as well as between tetragonal ZrO_2_ (112) and cubic Cu (311), is less than 4% (Figure 8). This facilitates the formation of a well-defined, extended Cu/tetragonal ZrO_2_ interface with superior CO_2_ selectivity in methanol steam reforming. The prevailing epitaxial relation is Cu(001)//tetragonal ZrO_2_ (112). Note that the discussed epitaxial Cu-tetragonal ZrO_2_ effects are very similar to those reported, e.g., for Au/rutile TiO_2_ in CO oxidation.^71^ For Cu/tetragonal ZrO_2_, the role of the initial hexagonal intermetallic compound Cu_51_Zr_14_ is central, as structural similarities between Cu_51_Zr_14_ and tetragnal ZrO_2_ additionally prevail. The dominating epitaxial relation between Cu_51_Zr_14_ and tetragonal ZrO_2_ is Cu_51_Zr_14_(0001)//tetragonal ZrO_2_ (112). The *in situ* decomposition of Cu_51_Zr_14_ is then directly steered by the energy gain of massively segregating and enriching metallic Cu at the surface, also facilitating the formation of well-ordered tetragonal ZrO_2_.

The role of tetragonal ZrO_2_ in CO_2_-selective methanol steam reforming on Cu catalysts^45^ reveals another very important feature: despite the prominent role of ZrO_2_, its use is severly hampered by the complex Zr–O phase diagram. At least three different crystalline ZrO_2_ polymorphs are known: the ambient-stable monoclinic modification and two high-temperature stable cubic and tetragonal structures.^72^ The latter two can be stabilized under ambient conditions by deliberate doping or particle size effects.^73^ Structural effects leading to high CO_2_ selectivity are only known for Cu in contact with tetragonal ZrO_2_, hence from the structural point of view, knowledge-based catalyst development should aim at providing synthesis pathways leading to maximum Cu-tetragonal ZrO_2_ phase boundary sites. The controlled *in situ* decomposition of Cu_51_Zr_14_ is one of the key preparation approaches to accomplish this, facilitated by the epitaxial relationships.

**Figure 9:**
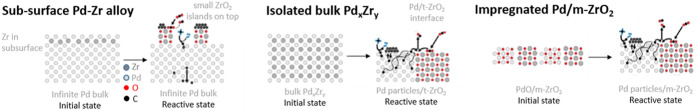
Schematic representation of the initial and reactive states of the three Pd–Zr materials. Reproduced with permission from ref (38). Copyright 2021 MDPI.

A similar metal-oxide phase boundary effect has been observed for a Pd_2_Zr intermetallic compound under methane dry reforming operation already discussed in the context of Figure 7.^23^ The exclusive activity-steering role of the Pd/ZrO_2_ interface can be directly appreciated by the comparison of the dry reforming reactivities of a surface Pd–Zr alloy, a Pd_2_Zr bulk intermetallic compound, and an impregnated Pd/ZrO_2_ catalyst.^38^ Mechanistic-wise, the extended Pd–ZrO_2_ interface present for the Pd_2_Zr intermetallic compound (and to a lesser extent for the impregnated Pd/ZrO_2_ material) allows efficient supply of the interface with reactive carbon arising from methane activation on metallic Pd (cf. Figure 7; Figure 9, middle and right panel).

Epitaxial relationships, as discussed in Figure 8, seem also to play a major role in the stabilization of the Pd/tetragonal ZrO_2_ interface during *in situ* activation of Pd_2_Zr in the methane dry reforming mixture, as the deviation in the lattice constant between Cu and Pd is only 7%.

### 3.1 Reactivity of the Resulting Oxide Polymorph

Elaborating on the importance of the metal–oxide phase boundary effects raises the question about the explicit structural and catalytic role of the oxide component as an integral part of the metal–oxide entity formed by *in situ* decomposition of the intermetallic compound/alloy. Apart from the general relevance of the oxide component for water activation in methanol steam reforming or carbon dioxide activation in methane dry reforming, the catalytic properties of the oxide can beneficially or detrimentally impact the total catalytic performance of the metal–oxide composite. To illustrate the general principles, we again turn to the group of Pd-based intermetallic compounds, specificially to the comparison of Zn–Pd, Ga–Pd, and In–Pd. The overall qualitative CO_2_ selectivity in the state after entering the intermetallic compound state following hydrogen reduction is comparable at >90%,^7,10,25^ but the minute differences can be also directly related to the intrinsic catalytic differences of ZnO, Ga_2_O_3_, and In_2_O_3_. The formation of ZnO and In_2_O_3_ patches on the (partially) decomposed ZnPd and InPd intermetallic compound has been directly proven by electron microscopy and directly correlated to an improved methanol steam reforming performance.^8,27,31^ Whether full decomposition of the intermetallic compound into a metal–oxide system or only a partial decomposition and the creation of an intermetallic compound/oxide interface occurs, the only important factor is that the nonoxidic component must be capable of efficient methanol activation. The CO_2_ selectivity of the resulting ZnPd/ZnO and InPd/In_2_O_3_ interfaces is more pronounced compared to Ga_2_Pd/Ga_2_O_3_.^8,22,24,26,27,31^ Especially for the InPd bimetallic catalysts, the synergy of the InPd bimetallic phase in contact with In_2_O_3_ has been documented for methanol steam reforming and methanol synthesis likewise.^31,74,75^

In contrast to ZnPd and InPd, the corresponding isolated Ga_2_Pd intermetallic compound is not susceptible to decomposition into Ga_2_Pd/Ga_2_O_3_.^30^ The only way to use Ga_2_Pd as an efficient methanol steam reforming catalyst is the preparation routine via reactive metal–support interaction, i.e., the reductive formation of GaPd_2_ particles by hydrogen reduction of a Pd/Ga_2_O_3_ catalyst at 773 K.^32^ The so-formed GaPd_2_/Ga_2_O_3_ interface is 95% CO_2_ selective in methanol steam reforming, which is less than that for ZnPd/ZnO (>99%) and InPd/In_2_O_3_ (>98%).^8,22,24,26,27,31,63^ The reason for this discrepancy is the pronounced water–gas shift reactivity of Ga_2_O_3_, which is spoiling the CO_2_ selectivity of the entire Ga_2_Pd/Ga_2_O_3_ catalyst at the methanol steam reforming reaction temperatures.^58,59^ Mechanistic-wise, the water–gas shift reaction on Ga_2_O_3_ can be purely surface-bound (formate-mediated) or involve oxygen vacancies (vacancy-mediated). ZnO and In_2_O_3_ are, however, very CO_2_-selective methanol steam reforming catalysts themselves, and the CO_2_ spoiling effect is efficiently suppressed.^11,57,60,61^ Especially on In_2_O_3_ as a highly reducible oxide, the water–gas shift route is purely oxygen vacancy-dominated (i.e., CO is very efficiently transformed into CO_2_), but the reverse reaction is effectively blocked due to the missing replenishment of oxygen vacancies by CO_2_.^57,60,61^

Exceeding the importance of the “simple” intrinsic catalytic properties of the oxide, the situation is significantly complicated by the fact that the adsorption and catalytic properties of a given oxide can be deliberately influenced by the synthesis protocol and steering the distribution of Brønsted and Lewis acidic and basic surface sites. Especially for intermetallic compounds involving Zr, eventually giving rise to ZrO_2_ during decomposition, this is a delicate issue. Although *in situ* decomposition of Cu-containing intermetallic compounds (Cu_51_Zr_14_ and CuZr_2_) yields a composite of metallic Cu and tetragonal ZrO_2_ due to the already discussed epitaxial stabilization, the previously anticipated exclusive role of tetragonal ZrO_2_ in CO_2_ selective methanol steam reforming cannot be upheld anymore. On the contrary, both ZrO_2_ modifications (monoclinic and tetragonal) can be switched between CO- and CO_2_-selective in contact with metallic Cu, depending on the surface acidity or basicity of ZrO_2_ as a consequence of the synthesis protocol.^76^

### 3.2 Carbon Reactivity

Carbon reactivity and clean-off as a key parameter for methane dry reforming activity is a direct consequence of the quality and quantity of the phase boundary sites (i.e., their activation capability and associated amount) arising from in situ decomposition of the intermetallic compound/alloy. As we have shown in comparative methane dry reforming studies using near-surface Pd–Zr alloys and bulk Pd_2_Zr intermetallic compounds,^23,38^ efficient carbon chemistry and loading can only be obtained on an extended Pd/tetragonal ZrO_2_ interface accessed through decomposition of the Pd_2_Zr intermetallic compound. Transfer of interfacial carbon as a consequence of methane activation on metallic Pd to redox-active ZrO_*x*_ sites assisting in CO_2_ activation is very efficient, and diffusive carbon loss into deeper Pd bulk regions is suppressed. Only starting from Pd_2_Zr yields the necessary small Pd particle dimensions for an increased amount of reactive carbide-like and/or dissolved carbon at the Pd-tetragonal ZrO_2_ phase boundary. The discussed carbon management, especially on Ni-containing intermetallic compounds, is very much related to the attempts to understand and accordingly suppress the coking on conventional Ni catalysts by active supports on a Zr- or La-oxide basis.^77,78^ “Active” support refers to the ability to decrease the carbon amount on the metal by usage of the phase boundary and the suppression of nucleation and formation of graphitic carbon layers also on the metal. Recent studies on Ni/MnO catalysts indicated that surface carbon can also act as a reactive intermediate under methane dry reforming operation but piles up as a significant amount of bulk carbon upon recooling to room temperature.^79^*In situ* characterization therefore is imperative to understand the carbon reactivity during catalytic operation, especially if intermetallic compounds are used as precursor structures to access the active metal-oxide phase. Decomposition of intermetallic compounds/alloys allows direct steerig of the dry reforming performance by optimization of the metal-oxide phase boundary and the associated metal particle size. In due course, the carbon dioxide activation properties, nucleation, and growth kinetics of graphite species or the role of reactive interfacial carbon can be influenced. As such, the requirements on the use and decomposition of intermetallic compounds in methane dry reforming are much higher compared to in methanol steam reforming, essentially due the carbon reactivity issue.

## 4 Conclusions and Outlook

We have shown the capabilities of using defined and ordered intermetallic compounds and alloys to prepare highly active and selective metal–oxide composite materials by *in situ* decomposition in the respective reaction mixtures. Exemplified for the methanol steam reforming and methane dry reforming reaction, we are able to identify a number of key factors that need to be carefully controlled to steer the decomposition pathway to catalytically prospective materials. The resulting quality (opening the desired reaction channels by selective activation) and associated large amounts of metal–oxide phase boundary sites is the single most important parameter that controls epitaxial relationships, the contribution of the intrinsic physicochemical/catalytic properties of the resulting oxide polymorph or the carbon reactivity. Thus, it determines the catalytic performance of the entire catalytic composite resulting from the *in situ* decomposition of intermetallic compound/alloy precursor structures. Appreciating the importance of the discussed key factors now allows projection of the performance of relevant catalytic materials beyond the exemplified case studies and eventual identification of similar materials on a knowledge-based basis. For methanol steam reforming, the prerequisite for an active and selective material is efficient methanol and water activation; therefore, it appears feasible to test the *in situ* decomposition of the respective group of intermetallic compounds on a copper basis, Cu–Ga,^80,81^ Cu–Sn,^82,83^ and Cu–Y;^79^ a palladium basis, Pd–Sn^84^ and Pd–Y;^85^ a platinum basis, Pt–Sn^86^ and Pt–Y;^87^ or an iridium basis, Ir–Ga,^88,89^ Ir–Sn,^88^ Ir–In,^90^ or Ir–Y.^91^ Intermetallic compounds exist in all binary phase diagrams. The selection of the “metal” part as Cu, Pd, Pt, or Ir is derived from the already documented methanol activation capabilities,^10,89^ the one for the “oxide” part from the known water activation capabilities of the oxide formed by the decomposition of the precursor structures.^11,57−59,92,93^ We expect the formation of Ga_2_O_3_, SnO_2_, and Y_2_O_3_ during decomposition—especially the latter two are proven to be highly CO_2_ selective methanol steam reforming catalysts themselves.^92−94^ Whether full decomposition to the metal–oxide systems or partial decomposition into oxide-supported intermetallic compounds, eventually through compositional intertransformations of different structures, occurs remains to be tested. In the best scenario, steering the decomposition process as a function of reaction temperature allows access to different structural stages of decomposition. The already tested Cu–In phase diagram is such a system, where through the combination of *in situ* decomposition studies of intermetallic compound precursor structures with different Cu/In ratios and impregnated Cu/In_2_O_3_ catalysts, the highly CO_2_ selective nature of the Cu–In_2_O_3_ interface was assessed.^95^

To extrapolate the use of *in situ* decomposition of Pd–Zr intermetallic compounds to access active methane dry reforming Pd-ZrO_2_ metal–oxide interfaces, its carbon management is crucial. For efficient bifunctional operation, it is necessary that the metal formed upon decomposition either forms a reactive carbide or actually dissolves carbon to yield a distinct carbon reactivity and allows for efficient carbon dioxide activation. The true nature of the activated carbon dioxide species, e.g., as intermediate (oxy)carbonate species, remains to be determined. The minimum requirement is that a full carbon dioxide activation–carbon monoxide release cycle must be enabled. This is particularly aided by basic surface sites, which have been documented to be crucial for CO_2_ activation and improvement of catalyst deactivation. For methane dry reforming, the addition of La_2_O_3_ to Co/SiO_2_ catalysts was reported to positively affect the surface basicity and catalytic properties.^96^ In due course, La-, Zr-, or Sm-containing intermetallic compounds represent a promising group as test structures, as—*in situ* decomposition of the intermetallic compounds provided—the resulting oxide parts La_2_O_3_, ZrO_2_, and Sm_2_O_3_ are already known from complementary studies on metal exsolution from perovskite-type oxides and intermetallic compounds during *in situ* dry reforming treatment to enable such a CO_2_ activation cycle.^38,77,97−100^ The decomposition of intermetallic compounds is a similar process insofar as an *in situ* formed metal-oxide interface is the active catalytic center. Intermetallic compounds such as binary Ni–La,^101,102^ Pd–La,^101^ or the corresponding Zr-^103,104^ or Sm-containing systems^100^ provide a reasonable starting point for *in situ* decomposition studies. The common reactivity denominator of Ni and Pd is the rich and vital carbon chemistry that has already been proven crucial for Pd–Zr systems, where only the *in situ* decomposition of Pd_2_Zr yielded the necessary active nanoparticulate Pd-ZrO_2_ composite.

Reaction-wise, we note that the concept, which was outlined for two examples, can be projected to related reactions. For CO and/or CO_2_ methanation and ammonia synthesis, such a concept was already introduced. A necessary prerequisite is that the oxidation/reduction chemical potential of the respective reaction mixture allows an approach to the stability limits of the intermetallic compound/alloy structures under the chosen reaction conditions.

As an important feature for full appreciation of the used concept, which has unfortunately not been touched so far, is related to the regeneration of the final metal-oxide composite mixture. This is of obvious importance for repeated use in catalytic cycles. If a full regeneration cycle can be repeatedly accessed and the final metal-oxide mixture can be obtained as a “steady state” of reversible decomposition and regeneration remains to be tested for each individual case. Attempts for such oxidative regeneration of In–Pd intermetallic compounds (which have been decomposed to Pd//In_2_O_3_ during activation) yielded unsatisfactory results. Although the reduced In_*x*_O_*y*_ could be restored, it forms a passivating layer around the Pd particles, preventing full regeneration of In–Pd.^27^ For UHV-based alloy model catalysts such as Cu–Zn or Zn–Pd discussed in this work, oxidative regeneration was possible by resegregation of Zn to the surface and associated removal.^35,55^

## References

[ref1] Armbrüster M. Intermetallic Compounds In Catalysis - A Versatile Class of Materials Meets Interesting Challenges. Sci. Technol. Adv. Mater. 2020, 21, 303–322. 10.1080/14686996.2020.1758544. 33628119 PMC7889166

[ref2] Armbrüster M.; Schlögl R.; Grin Y. Intermetallic Compounds In Heterogeneous Catalysis—A Quickly Developing Field. Sci. Technol. Adv. Mater. 2014, 15, 034803 10.1088/1468-6996/15/3/034803. 27877674 PMC5090519

[ref3] Dasgupta A.; Rioux R. M. Intermetallics In Catalysis: An Exciting Subset of Multimetallic Catalysts. Catal. Today 2019, 330, 2–15. 10.1016/j.cattod.2018.05.048.

[ref4] Furukawa S.; Komatsu T. Intermetallic Compounds: Promising Inorganic Materials for Well-Structured and Electronically Modified Reaction Environments for Efficient Catalysis. ACS Catal. 2017, 7, 735–765. 10.1021/acscatal.6b02603.

[ref5] Marakatti V. S.; Peter S. C. Synthetically Tuned Electronic and Geometrical Properties of Intermetallic Compounds As Effective Heterogeneous Catalysts. Prog. Solid State Chem. 2018, 52, 1–30. 10.1016/j.progsolidstchem.2018.09.001.

[ref6] Rößner L.; Armbrüster M. Electrochemical Energy Conversion on Intermetallic Compounds: A Review. ACS Catal. 2019, 9, 2018–2062. 10.1021/acscatal.8b04566.

[ref7] Armbrüster M.; Behrens M.; Föttinger K.; Friedrich M.; Gaudry É.; Matam S. K.; Sharma H. R. The Intermetallic Compound ZnPd and Its Role in Methanol Steam Reforming. Catal. Rev.: Sci. Eng. 2013, 55, 289–367. 10.1080/01614940.2013.796192.

[ref8] Friedrich M.; Penner S.; Heggen M.; Armbrüster M. High CO_2_ Selectivity in Methanol Steam Reforming through ZnPd/ZnO Teamwork. Angew. Chem., Int. Ed. 2013, 52, 4389–4392. 10.1002/anie.201209587. 23494806

[ref9] Friedrich M.; Teschner D.; Knop-Gericke A.; Armbrüster M. Influence of Bulk Composition of The Intermetallic Compound ZnPd on Surface Composition and Methanol Steam Reforming Properties. J. Catal. 2012, 285, 41–47. 10.1016/j.jcat.2011.09.013.

[ref10] Iwasa N.; Takezawa N. New Supported Pd and Pt Alloy Catalysts for Steam Reforming and Dehydrogenation of Methanol. Top. Catal. 2003, 22, 215–224. 10.1023/A:1023571819211.

[ref11] Lorenz H.; Friedrich M.; Armbrüster M.; Klötzer B.; Penner S. ZnO Is a CO_2_-Selective Steam Reforming Catalyst. J. Catal. 2013, 297, 151–154. 10.1016/j.jcat.2012.10.003. 23335817 PMC3546163

[ref12] Takeshita T.; Wallace W. E.; Craig R. S. Rare Earth Intermetallics As Synthetic Ammonia Catalysts. J. Catal. 1976, 44, 236–243. 10.1016/0021-9517(76)90394-8.

[ref13] Elattar A.; Wallace W. E.; Craig R. S. Hydrocarbon Synthesis Using Catalysts Formed by Intermetallic Compound Decomposition. In Hydrocarbon Synthesis from Carbon Monoxide and Hydrogen; American Chemical Society, 1979; Vol. 178; pp 7–14. 10.1021/ba-1979-0178.ch002.

[ref14] Coon V. T.; Takeshita T.; Wallace W. E.; Craig R. S. Rare Earth Intermetallics as Catalysts For The Production of Hydrocarbons From Carbon Monoxide and Hydrogen. J. Phys. Chem. 1976, 80, 1878–1879. 10.1021/j100558a013.

[ref15] Elattar A.; Takeshita T.; Wallace W.; Craig R. Intermetallic Compounds of The Type MNi5 as Methanation Catalysts. Science 1977, 196, 1093–1094. 10.1126/science.196.4294.1093. 17778546

[ref16] Zhang Z.; Verykios X. E.; MacDonald S. M.; Affrossman S. Comparative Study of Carbon Dioxide Reforming of Methane to Synthesis Gas over Ni/La_2_O_3_ and Conventional Nickel-Based Catalysts. J. Phys. Chem. 1996, 100, 744–754. 10.1021/jp951809e.

[ref17] Moldovan A.; Elattar A.; Wallace W. Studies of the Methanation Catalysts ThNi5 and ZrNi5 by Auger and Characteristic Energy Loss Spectra. J. Solid State Chem. 1978, 25, 23–29. 10.1016/0022-4596(78)90039-7.

[ref18] Baiker A.; Schlog̈l R.; Armbruster E.; Güntherodt H. J. Ammonia Synthesis over Supported Iron Catalyst Prepared From Amorphous Iron-Zirconium Precursor: I. Bulk Structural and Surface Chemical Changes of Precursor During Its Transition to The Active Catalyst. J. Catal. 1987, 107, 221–231. 10.1016/0021-9517(87)90287-9.

[ref19] Noack K.; Rehren C.; Zbinden H.; Schloegl R. Modification of the Catalytic Hydrogenation Activity of Glassy Pd81Si19. Surface Analysis by ISS and XPS. Langmuir 1995, 11, 2018–2030. 10.1021/la00006a031.

[ref20] Baiker A.; Gasser D.; Lenzner J.; Reller A.; Schlogl R. Oxidation of Carbon Monoxide over Palladium on Zirconia Prepared from Amorphous Pd—Zr Alloy I. Bulk Structural, Morphological, and Catalytic Properties of Catalyst. J. Catal. 1990, 126, 555–571. 10.1016/0021-9517(90)90020-K.

[ref21] Tsai A. P.; Yoshimura M. Highly Active Quasicrystalline Al-Cu-Fe Catalyst For Steam Reforming of Methanol. Appl. Catal., A 2001, 214, 237–241. 10.1016/S0926-860X(01)00500-2.

[ref22] Haghofer A.; Föttinger K.; Girgsdies F.; Teschner D.; Knop-Gericke A.; Schlögl R.; Rupprechter G. In Situ Study of The Formation And Stability of Supported Pd_2_Ga Methanol Steam Reforming Catalysts. J. Catal. 2012, 286, 13–21. 10.1016/j.jcat.2011.10.007.

[ref23] Köpfle N.; Mayr L.; Schmidmair D.; Bernardi J.; Knop-Gericke A.; Hävecker M.; Klötzer B.; Penner S. A Comparative Discussion of the Catalytic Activity and CO_2_-Selectivity of Cu-Zr and Pd-Zr (Intermetallic) Compounds in Methanol Steam Reforming. Catalysts 2017, 7, 53 10.3390/catal7020053.

[ref24] Lorenz H.; Penner S.; Jochum W.; Rameshan C.; Klötzer B. Pd/Ga_2_O_3_ Methanol Steam Reforming Catalysts: Part II. Catalytic Selectivity. Appl. Catal., A 2009, 358, 203–210. 10.1016/j.apcata.2009.02.027.

[ref25] Lorenz H.; Rameshan C.; Bielz T.; Memmel N.; Stadlmayr W.; Mayr L.; Zhao Q.; Soisuwan S.; Klötzer B.; Penner S. From Oxide-Supported Palladium to Intermetallic Palladium Phases: Consequences for Methanol Steam Reforming. ChemCatChem 2013, 5, 1273–1285. 10.1002/cctc.201200712.

[ref26] Lorenz H.; Thalinger R.; Köck E.-M.; Kogler M.; Mayr L.; Schmidmair D.; Bielz T.; Pfaller K.; Klötzer B.; Penner S. Methanol Steam Reforming: CO_2_-Selective Pd_2_Ga Phases Supported on α- and γ-Ga_2_O_3_. Appl. Catal., A 2013, 453, 34–44. 10.1016/j.apcata.2012.11.010.

[ref27] Lorenz H.; Turner S.; Lebedev O. I.; Van Tendeloo G.; Klötzer B.; Rameshan C.; Pfaller K.; Penner S. Pd-In_2_O_3_ Interaction Due to Reduction In Hydrogen: Consequences for Methanol Steam Reforming. Appl. Catal., A 2010, 374, 180–188. 10.1016/j.apcata.2009.12.007.

[ref28] Mayr L.; Klötzer B.; Schmidmair D.; Köpfle N.; Bernardi J.; Schwarz S.; Armbrüster M.; Penner S. Boosting Hydrogen Production from Methanol and Water by In Situ Activation of Bimetallic Cu-Zr Species. ChemCatChem 2016, 8, 1778–1781. 10.1002/cctc.201600361.

[ref29] Mayr L.; Köpfle N.; Klötzer B.; Götsch T.; Bernardi J.; Schwarz S.; Keilhauer T.; Armbrüster M.; Penner S. Microstructural and Chemical Evolution and Analysis of a Self-Activating CO_2_-Selective Cu-Zr Bimetallic Methanol Steam Reforming Catalyst. J. Phys. Chem. C 2016, 120, 25395–25404. 10.1021/acs.jpcc.6b07824.

[ref30] Mayr L.; Lorenz H.; Armbruster M.; Villaseca S. A.; Luo Y.; Cardoso R.; Burkhardt U.; Zemlyanov D.; Haevecker M.; Blume R.; Knop-Gericke A.; Klotzer B.; Penner S. The Catalytic Properties of Thin Film Pd-Rich GaPd_2_ In Methanol Steam Reforming. J. Catal. 2014, 309, 231–240. 10.1016/j.jcat.2013.10.002.

[ref31] Neumann M.; Teschner D.; Knop-Gericke A.; Reschetilowski W.; Armbrüster M. Controlled Synthesis and Catalytic Properties of Supported In-Pd Intermetallic Compounds. J. Catal. 2016, 340, 49–59. 10.1016/j.jcat.2016.05.006.

[ref32] Penner S.; Armbrüster M. Formation of Intermetallic Compounds by Reactive Metal-Support Interaction: A Frequently Encountered Phenomenon In Catalysis. ChemCatChem 2015, 7, 374–392. 10.1002/cctc.201402635.

[ref33] Penner S.; Lorenz H.; Jochum W.; Stöger-Pollach M.; Wang D.; Rameshan C.; Klötzer B. Pd/Ga_2_O_3_ Methanol Steam Reforming Catalysts: Part I. Morphology, Composition and Structural Aspects. Appl. Catal., A 2009, 358, 193–202. 10.1016/j.apcata.2009.02.026.

[ref34] Rameshan C.; Stadlmayr W.; Penner S.; Lorenz H.; Mayr L.; Hävecker M.; Blume R.; Rocha T.; Teschner D.; Knop-Gericke A.; Schlögl R.; Zemlyanov D.; Memmel N.; Klötzer B. In Situ XPS Study of Methanol Reforming on PdGa Near-Surface Intermetallic Phases. J. Catal. 2012, 290, 126–137. 10.1016/j.jcat.2012.03.009. 22875996 PMC3405296

[ref35] Rameshan C.; Stadlmayr W.; Weilach C.; Penner S.; Lorenz H.; Hävecker M.; Blume R.; Rocha T.; Teschner D.; Knop-Gericke A.; Schlögl R.; Memmel N.; Zemlyanov D.; Rupprechter G.; Klötzer B. Subsurface-Controlled CO_2_ Selectivity of PdZn Near-Surface Alloys in H_2_ Generation by Methanol Steam Reforming. Angew. Chem., Int. Ed. 2010, 49, 3224–3227. 10.1002/anie.200905815. 20352638

[ref36] Furukawa S.; Endo M.; Komatsu T. Bifunctional Catalytic System Effective for Oxidative Dehydrogenation of 1-Butene and n-Butane Using Pd-Based Intermetallic Compounds. ACS Catal. 2014, 4, 3533–3542. 10.1021/cs500920p.

[ref37] Köpfle N.; Götsch T.; Grünbacher M.; Carbonio E. A.; Hävecker M.; Knop-Gericke A.; Schlicker L.; Doran A.; Kober D.; Gurlo A.; Penner S.; Klötzer B. Zirconium-Assisted Activation of Palladium To Boost Syngas Production by Methane Dry Reforming. Angew. Chem., Int. Ed. 2018, 57, 14613–14618. 10.1002/anie.201807463. PMC6221108 30179293

[ref38] Köpfle N.; Ploner K.; Lackner P.; Götsch T.; Thurner C.; Carbonio E.; Hävecker M.; Knop-Gericke A.; Schlicker L.; Doran A.; Kober D.; Gurlo A.; Willinger M.; Penner S.; Schmid M.; Klötzer B. Carbide-Modified Pd on ZrO_2_ as Active Phase for CO_2_-Reforming of Methane—A Model Phase Boundary Approach. Catalysts 2020, 10, 1000 10.3390/catal10091000.

[ref39] Shoji S.; Peng X.; Imai T.; Murphin Kumar P. S.; Higuchi K.; Yamamoto Y.; Tokunaga T.; Arai S.; Ueda S.; Hashimoto A.; Tsubaki N.; Miyauchi M.; Fujita T.; Abe H. Topologically immobilized catalysis centre for long-term stable carbon dioxide reforming of methane. Chem. Sci. 2019, 10, 3701–3705. 10.1039/C8SC04965C. 31015913 PMC6461125

[ref40] Komatsu T.; Uezono T. CO_2_ Reforming of Methane on Ni- and Co-based Intermetallic Compound Catalysts. J. Jpn. Pet. Inst. 2005, 48, 76–83. 10.1627/jpi.48.76.

[ref41] Baiker A. Metallic Glasses In Heterogeneous Catalysis. Faraday Discuss. Chem. Soc. 1989, 87, 239–251. 10.1039/dc9898700239.

[ref42] Takahashi T.; Inoue M.; Kai T. Effect of Metal Composition on Hydrogen Selectivity In Steam Reforming of Methanol over Catalysts Prepared From Amorphous Alloys. Appl. Catal., A 2001, 218, 189–195. 10.1016/S0926-860X(01)00641-X.

[ref43] Pang Tsai A.; Kameoka S.; Ishii Y. PdZn = Cu: Can an Intermetallic Compound Replace an Element?. J. Phys. Soc. Jpn. 2004, 73, 3270–3273. 10.1143/JPSJ.73.3270.

[ref44] Breen J. P.; Ross J. R. H. Methanol Reforming For Fuel-Cell Applications: Development of Zirconia-Containing Cu-Zn-Al Catalysts. Catal. Today 1999, 51, 521–533. 10.1016/S0920-5861(99)00038-3.

[ref45] Purnama H.; Girgsdies F.; Ressler T.; Schattka J. H.; Caruso R. A.; Schomäcker R.; Schlögl R. Activity and Selectivity of a Nanostructured CuO/ZrO_2_ Catalyst in the Steam Reforming of Methanol. Catal. Lett. 2004, 94, 61–68. 10.1023/B:CATL.0000019332.80287.6b.

[ref46] Velu S.; Suzuki K.; Gopinath C. S.; Yoshida H.; Hattori T. XPS, XANES and EXAFS Investigations of CuO/ZnO/Al_2_O_3_/ZrO_2_ Mixed Oxide Catalysts. Phys. Chem. Chem. Phys. 2002, 4, 1990–1999. 10.1039/b109766k.

[ref47] Velu S.; Suzuki K.; Kapoor M. P.; Ohashi F.; Osaki T. Selective Production of Hydrogen For Fuel Cells Via Oxidative Steam Reforming of Methanol over CuZnAl(Zr)-Oxide Catalysts. Appl. Catal., A 2001, 213, 47–63. 10.1016/S0926-860X(00)00879-6.

[ref48] Gasser D.; Baiker A. Hydrogenation of Carbon Dioxide Over Copper—Zirconia Catalysts Prepared by In-Situ Activation of Amorphous Copper—Zirconium Alloy. Appl. Catal. 1989, 48, 279–294. 10.1016/S0166-9834(00)82799-2.

[ref49] Domokos L.; Katona T.; Molnár Á.; Lovas A. Amorphous Alloy Catalysis VIII. A New Activation of An Amorphous Cu_41_Zr_59_ Alloy In The Transformation of Methyl Alcohol To Methyl Formate. Appl. Catal., A 1996, 142, 151–158. 10.1016/0926-860X(96)00051-8.

[ref50] Jennings J. R.; Owen G.; Nix R. M.; Lambert R. M. Methanol Synthesis Catalysts Derived From Copperintermetallic Precursors: Transient Response to Pulses of Carbon Dioxide, Oxygen And Nitrous Oxide. Appl. Catal., A 1992, 82, 65–75. 10.1016/0926-860X(92)80006-X.

[ref51] Owen G.; Hawkes C. M.; Lloyd D.; Jennings J. R.; Lambert R. M.; Nix R. M. Methanol Synthesis Catalysts Derived From Ternary Rare Earth, Copper, Zirconium And Rare Earth, Copper, Titanium Intermetallic Alloys. Appl. Catal. 1990, 58, 69–81. 10.1016/S0166-9834(00)82279-4.

[ref52] Chase M. W. Jr. NIST-JANAF Themochemical Tables, Fourth Edition. J. Phys. Chem. Ref. Data 1998, 9, 1–1951. Monograph.

[ref53] Mayr L.; Klötzer B.; Zemlyanov D.; Penner S. Steering of Methanol Reforming Selectivity By Zirconia-Copper Interaction. J. Catal. 2015, 321, 123–132. 10.1016/j.jcat.2014.10.012.

[ref54] Mayr L.; Shi X.; Köpfle N.; Klötzer B.; Zemlyanov D. Y.; Penner S. Tuning of The Copper-Zirconia Phase Boundary For Selectivity Control of Methanol Conversion. J. Catal. 2016, 339, 111–122. 10.1016/j.jcat.2016.03.029.

[ref55] Rameshan C.; Stadlmayr W.; Penner S.; Lorenz H.; Memmel N.; Hävecker M.; Blume R.; Teschner D.; Rocha T.; Zemlyanov D.; Knop-Gericke A.; Schlögl R.; Klötzer B. Hydrogen Production by Methanol Steam Reforming on Copper Boosted by Zinc-Assisted Water Activation. Angew. Chem., Int. Ed. 2012, 51, 3002–3006. 10.1002/anie.201106591. PMC3556650 22337500

[ref56] Penner S.; Wang D.; Su D. S.; Rupprechter G.; Podloucky R.; Schlögl R.; Hayek K. Platinum Nanocrystals Supported by Silica, Alumina And Ceria: Metal-Support Interaction Due to High-Temperature Reduction In Hydrogen. Surf. Sci. 2003, 532, 276–280. 10.1016/S0039-6028(03)00198-5.

[ref57] Lorenz H.; Jochum W.; Klötzer B.; Stöger-Pollach M.; Schwarz S.; Pfaller K.; Penner S. Novel Methanol Steam Reforming Activity and Selectivity of Pure In_2_O_3_. Appl. Catal., A 2008, 347, 34–42. 10.1016/j.apcata.2008.05.028.

[ref58] Jochum W.; Penner S.; Föttinger K.; Kramer R.; Rupprechter G.; Klötzer B. Hydrogen on Polycrystalline β-Ga_2_O_3_: Surface Chemisorption, Defect Formation, And Reactivity. J. Catal. 2008, 256, 268–277. 10.1016/j.jcat.2008.03.019.

[ref59] Jochum W.; Penner S.; Kramer R.; Föttinger K.; Rupprechter G.; Klötzer B. Defect Formation and The Water-Gas Shift Reaction on B-Ga_2_O_3_. J. Catal. 2008, 256, 278–286. 10.1016/j.jcat.2008.03.018.

[ref60] Bielz T.; Lorenz H.; Amann P.; Klötzer B.; Penner S. Water-Gas Shift and Formaldehyde Reforming Activity Determined by Defect Chemistry of Polycrystalline In_2_O_3_. J. Phys. Chem. C 2011, 115, 6622–6628. 10.1021/jp111739m.

[ref61] Bielz T.; Lorenz H.; Jochum W.; Kaindl R.; Klauser F.; Klötzer B.; Penner S. Hydrogen on In_2_O_3_: Reducibility, Bonding, Defect Formation, and Reactivity. J. Phys. Chem. C 2010, 114, 9022–9029. 10.1021/jp1017423.

[ref62] Heggen M.; Penner S.; Friedrich M.; Dunin-Borkowski R. E.; Armbrüster M. Formation of ZnO Patches on ZnPd/ZnO during Methanol Steam Reforming: A Strong Metal-Support Interaction Effect?. J. Phys. Chem. C 2016, 120, 10460–10465. 10.1021/acs.jpcc.6b02562.

[ref63] Haghofer A.; Ferri D.; Föttinger K.; Rupprechter G. Who Is Doing the Job? Unraveling the Role of Ga_2_O_3_ in Methanol Steam Reforming on Pd_2_Ga/Ga_2_O_3_. ACS Catal. 2012, 2, 2305–2315. 10.1021/cs300480c.

[ref64] Ghoneim S. A.; El-Salamony R. A.; El-Temtamy S. A. Review on Innovative Catalytic Reforming of Natural Gas to Syngas. World J. Eng. Technol. 2016, 4, 116 10.4236/wjet.2016.41011.

[ref65] Wang S.; Lu G. Q.; Millar G. J. Carbon Dioxide Reforming of Methane To Produce Synthesis Gas over Metal-Supported Catalysts: State of the Art. Energy Fuels 1996, 10, 896–904. 10.1021/ef950227t.

[ref66] Ashcroft A. T.; Cheetham A. K.; Green M. L. H.; Vernon P. D. F. Partial Oxidation of Methane to Synthesis Gas Using Carbon Dioxide. Nature 1991, 352, 225–226. 10.1038/352225a0.

[ref67] Bradford M. C. J.; Vannice M. A. Catalytic Reforming of Methane With Carbon Dioxide over Nickel Catalysts I. Catalyst Characterization and Activity. Appl. Catal., A 1996, 142, 73–96. 10.1016/0926-860X(96)00065-8.

[ref68] Rostrupnielsen J. R.; Hansen J. H. B. CO_2_-Reforming of Methane over Transition Metals. J. Catal. 1993, 144, 38–49. 10.1006/jcat.1993.1312.

[ref69] Wolfbeisser A.; Sophiphun O.; Bernardi J.; Wittayakun J.; Föttinger K.; Rupprechter G. Methane Dry Reforming over Ceria-Zirconia Supported Ni Catalysts. Catal. Today 2016, 277, 234–245. 10.1016/j.cattod.2016.04.025.

[ref70] Steinhauer B.; Kasireddy M. R.; Radnik J.; Martin A. Development of Ni-Pd Bimetallic Catalysts For The Utilization of Carbon Dioxide and Methane by Dry Reforming. Appl. Catal., A 2009, 366, 333–341. 10.1016/j.apcata.2009.07.021.

[ref71] Haruta M. When Gold Is Not Noble: Catalysis by Nanoparticles. Chem. Rec. 2003, 3, 75–87. 10.1002/tcr.10053. 12731078

[ref72] Abriata J. P.; Garcés J.; Versaci R. The O-Zr (Oxygen-Zirconium) System. Bull. Alloy Phase Diagrams 1986, 7, 116–124. 10.1007/BF02881546.

[ref73] Shukla S.; Seal S. Mechanisms of Room Temperature Metastable Tetragonal Phase Stabilisation In Zirconia. Int. Mater. Rev. 2005, 50, 45–64. 10.1179/174328005X14267.

[ref74] Köwitsch N.; Thoni L.; Klemmed B.; Benad A.; Paciok P.; Heggen M.; Köwitsch I.; Mehring M.; Eychmüller A.; Armbrüster M. Proving a Paradigm in Methanol Steam Reforming: Catalytically Highly Selective In_x_Pd_y_/In_2_O_3_ Interfaces. ACS Catal. 2021, 11, 304–312. 10.1021/acscatal.0c04073.

[ref75] Snider J. L.; Streibel V.; Hubert M. A.; Choksi T. S.; Valle E.; Upham D. C.; Schumann J.; Duyar M. S.; Gallo A.; Abild-Pedersen F.; Jaramillo T. F. Revealing the Synergy between Oxide and Alloy Phases on the Performance of Bimetallic In-Pd Catalysts for CO_2_ Hydrogenation to Methanol. ACS Catal. 2019, 9, 3399–3412. 10.1021/acscatal.8b04848.

[ref76] Ploner K.; Watschinger M.; Kheyrollahi Nezhad P. D.; Götsch T.; Schlicker L.; Köck E.-M.; Gurlo A.; Gili A.; Doran A.; Zhang L.; Köwitsch N.; Armbrüster M.; Vanicek S.; Wallisch W.; Thurner C.; Klötzer B.; Penner S. Mechanistic Insights Into The Catalytic Methanol Steam Reforming Performance of Cu/ZrO_2_ Catalysts By In Situ and Operando Studies. J. Catal. 2020, 391, 497–512. 10.1016/j.jcat.2020.09.018.

[ref77] Bekheet M. F.; Delir Kheyrollahi Nezhad P.; Bonmassar N.; Schlicker L.; Gili A.; Praetz S.; Gurlo A.; Doran A.; Gao Y.; Heggen M.; Niaei A.; Farzi A.; Schwarz S.; Bernardi J.; Klötzer B.; Penner S. Steering the Methane Dry Reforming Reactivity of Ni/La_2_O_3_ Catalysts by Controlled In Situ Decomposition of Doped La_2_NiO_4_ Precursor Structures. ACS Catal. 2021, 11, 43–59. 10.1021/acscatal.0c04290. 33425477 PMC7783868

[ref78] Liu W.; Li L.; Zhang X.; Wang Z.; Wang X.; Peng H. Design of Ni-ZrO_2_@SiO_2_ Catalyst with Ultra-High Sintering and Coking Resistance For Dry Reforming of Methane to Prepare Syngas. J. CO_2_ Util. 2018, 27, 297–307. 10.1016/j.jcou.2018.08.003.

[ref79] Gili A.; Schlicker L.; Bekheet M. F.; Görke O.; Penner S.; Grünbacher M.; Götsch T.; Littlewood P.; Marks T. J.; Stair P. C.; Schomäcker R.; Doran A.; Selve S.; Simon U.; Gurlo A. Surface Carbon as a Reactive Intermediate in Dry Reforming of Methane to Syngas on a 5% Ni/MnO Catalyst. ACS Catal. 2018, 8, 8739–8750. 10.1021/acscatal.8b01820.

[ref80] Liu S.; McDonald S.; Gu Q.; Matsumura S.; Qu D.; Sweatman K.; Nishimura T.; Nogita K. Properties of CuGa_2_ Formed Between Liquid Ga and Cu Substrates at Room Temperature. J. Electron. Mater. 2020, 49, 128–139. 10.1007/s11664-019-07688-4.

[ref81] Li J.-B.; Ji L.N.; Liang J.K.; Zhang Y.; Luo J.; Li C.R.; Rao G.H. A Thermodynamic Assessment of the Copper-Gallium System. CALPHAD: Comput. Coupling Phase Diagrams Thermochem. 2008, 32, 447–453. 10.1016/j.calphad.2008.03.006.

[ref82] Li D.; Franke P.; Fürtauer S.; Cupid D.; Flandorfer H. The Cu-Sn Phase Diagram Part II: New Thermodynamic Assessment. Intermetallics 2013, 34, 148–158. 10.1016/j.intermet.2012.10.010. PMC4819024 27087755

[ref83] Fürtauer S.; Li D.; Cupid D.; Flandorfer H. The Cu-Sn Phase Diagram, Part I: New Experimental Results. Intermetallics 2013, 34, 142–147. 10.1016/j.intermet.2012.10.004. 27087755 PMC4819024

[ref84] Okamoto H. Pd-Sn (Palladium-Tin). J. Phase Equilib. Diffus. 2012, 33, 253–254. 10.1007/s11669-012-0025-0.

[ref85] Du Z.; Yang H.; Li C. Thermodynamic Modeling of the Pd-Y System. J. Alloys Compd. 2000, 297, 185–191. 10.1016/S0925-8388(99)00582-4.

[ref86] Okamoto H. The Pt-Sn (Platinum-Tin) System. J. Phase Equilib. 2003, 24, 198–198. 10.1361/105497103770330938.

[ref87] Palenzona A.; Cirafici S. The Pt-Y (Platinum-Yttrium) System. J. Phase Equilib. 1990, 11, 493–497. 10.1007/BF02898267.

[ref88] Ir (Iridium) Binary Alloy Phase Diagrams. In Alloy Phase Diagrams; Okamoto H., Schlesinger M. E., Mueller E. M., Eds.; ASM International, 2016; Vol. 3, p 1, 10.31399/asm.hb.v03.a0006172.

[ref89] Weststrate C. J.; Ludwig W.; Bakker J. W.; Gluhoi A. C.; Nieuwenhuys B. E. Methanol Decomposition and Oxidation on Ir(111). J. Phys. Chem. C 2007, 111, 7741–7747. 10.1021/jp070539k. 17366645

[ref90] Anres P.; Fossati P.; Richter K.; Gambino M.; Gaune-Escard M.; Bros J. P. Thermodynamics of the [Ir-In] System. J. Alloys Compd. 2000, 296, 119–127. 10.1016/S0925-8388(99)00507-1.

[ref91] Okamoto H. The Ir-Y (Iridium-Yttrium) System. J. Phase Equilib. 1992, 13, 651–653. 10.1007/BF02667218.

[ref92] Kuo H.-T.; Chen H.-W. Effect of Y_2_O_3_ and Nd_2_O_3_ on the Steam Reforming of Methanol over Cu/ZnO Catalysts. Sci. Adv. Mater. 2013, 5, 1895–1906. 10.1166/sam.2013.1655.

[ref93] Zhao Q.; Lorenz H.; Turner S.; Lebedev O. I.; Van Tendeloo G.; Rameshan C.; Klötzer B.; Konzett J.; Penner S. Catalytic Characterization of Pure SnO_2_ and GeO_2_ In Methanol Steam Reforming. Appl. Catal., A 2010, 375, 188–195. 10.1016/j.apcata.2009.12.027.

[ref94] Lorenz H.; Zhao Q.; Turner S.; Lebedev O. I.; Van Tendeloo G.; Klötzer B.; Rameshan C.; Pfaller K.; Konzett J.; Penner S. Origin of Different Deactivation of Pd/SnO_2_ and Pd/GeO_2_ Catalysts In Methanol Dehydrogenation and Reforming: A Comparative Study. Appl. Catal., A 2010, 381, 242–252. 10.1016/j.apcata.2010.04.015.

[ref95] Ploner K.; Schlicker L.; Gili A.; Gurlo A.; Doran A.; Zhang L.; Armbrüster M.; Obendorf D.; Bernardi J.; Klötzer B.; Penner S. Reactive Metal-Support Interaction In The Cu-In_2_O_3_ System: Intermetallic Compound Formation and Its Consequences For CO_2_-Selective Methanol Steam Reforming. Sci. Technol. Adv. Mater. 2019, 20, 356–366. 10.1080/14686996.2019.1590127. 31068984 PMC6493314

[ref96] Bouarab R.; Cherifi O.; Auroux A. Effect of The Basicity Created By La_2_O_3_ Addition on The Catalytic Properties of Co(O)/SiO_2_ In CH_4_+CO_2_ Reaction. Thermochim. Acta 2005, 434, 69–73. 10.1016/j.tca.2005.01.019.

[ref97] Osazuwa O. U.; Cheng C. K. Catalytic Conversion of Methane And Carbon Dioxide (Greenhouse Gases) Into Syngas over Samarium-Cobalt-Trioxides Perovskite Catalyst. J. Cleaner Prod. 2017, 148, 202–211. 10.1016/j.jclepro.2017.01.177.

[ref98] Osazuwa O. U.; Setiabudi H. D.; Abdullah S.; Cheng C. K. RETRACTED: Syngas Production From Methane Dry Reforming over SmCoO_3_ Perovskite Catalyst: Kinetics and Mechanistic Studies. Int. J. Hydrogen Energy 2017, 42, 9707–9721. 10.1016/j.ijhydene.2017.03.061.

[ref99] Zhang W. D.; Liu B. S.; Zhan Y. P.; Tian Y. L. Syngas Production via CO_2_ Reforming of Methane over Sm_2_O_3_-La_2_O_3_-Supported Ni Catalyst. Ind. Eng. Chem. Res. 2009, 48, 7498–7504. 10.1021/ie9001298.

[ref100] Taherian Z.; Yousefpour M.; Tajally M.; Khoshandam B. A Comparative Study of ZrO_2_, Y_2_O_3_ and Sm_2_O_3_ Promoted Ni/SBA-15 Catalysts For Evaluation of CO_2_/Methane Reforming Performance. Int. J. Hydrogen Energy 2017, 42, 16408–16420. 10.1016/j.ijhydene.2017.05.095.

[ref101] La (Lanthanum) Binary Alloy Phase Diagrams. Alloy Phase Diagrams; Okamoto H., Schlesinger M. E., Mueller E. M., Eds.; ASM International, 2016; Vol. 3, p 1. 10.31399/asm.hb.v03.a0006173.

[ref102] Deyuan Z.; Tang J.; Gschneidner K. A. A Redetermination of The La-Ni Phase Diagram From LaNi To LaNi_5_ (50–83.3 at.% Ni). J. Less-Common Met. 1991, 169, 45–53. 10.1016/0022-5088(91)90234-U.

[ref103] Pd (Palladium) Binary Alloy Phase Diagrams. Alloy Phase Diagrams; Okamoto H., Schlesinger M. E., Mueller E. M., Eds.; ASM International, 2016; Vol. 3, p 1. 10.31399/asm.hb.v03.a0006193.

[ref104] Su X.; Zhang W.; Du Z. A Thermodynamic Assessment of the Ni-Sm System. J. Alloys Compd. 1998, 278, 182–184. 10.1016/S0925-8388(98)00560-X.

